# Microglial cathepsin E plays a role in neuroinflammation and amyloid β production in Alzheimer’s disease

**DOI:** 10.1111/acel.13565

**Published:** 2022-02-19

**Authors:** Zhen Xie, Jie Meng, Wei Kong, Zhou Wu, Fei Lan, Narengaowa, Yoshinori Hayashi, Qinghu Yang, Zhantao Bai, Hiroshi Nakanishi, Hong Qing, Junjun Ni

**Affiliations:** ^1^ Key Laboratory of Molecular Medicine and Biotherapy Department of Biology School of Life Science Beijing Institute of Technology Beijing China; ^2^ Department of Neurology and State Key Laboratory of Biotherapy Collaborative Innovation Center for Biotherapy West China Hospital Sichuan University Chengdu China; ^3^ Department of Aging Science and Pharmacology Faculty of Dental Science Kyushu University Fukuoka Japan; ^4^ Department of Physiology Nihon University School of Dentistry Tokyo Japan; ^5^ Research Center for Resource Peptide Drugs Shaanxi Engineering & Technological Research Center for Conversation & Utilization of Regional Biological Resources Yan’an University Yan’an China; ^6^ Department of Pharmacology Faculty of Pharmacy Yasuda Women’s University Hiroshima Japan

**Keywords:** Alzheimers disease, amyloid‐β, cathepsin E, microglia, neuroinflammation, TRAIL

## Abstract

Regulation of neuroinflammation and β‐amyloid (Aβ) production are critical factors in the pathogenesis of Alzheimer's disease (AD). Cathepsin E (CatE), an aspartic protease, is widely studied as an inducer of growth arrest and apoptosis in several types of cancer cells. However, the function of CatE in AD is unknown. In this study, we demonstrated that the ablation of CatE in human amyloid precursor protein knock‐in mice, called APP^NL−G−F^ mice, significantly reduced Aβ accumulation, neuroinflammation, and cognitive impairments. Mechanistically, microglial CatE is involved in the secretion of soluble TNF‐related apoptosis‐inducing ligand, which plays an important role in microglia‐mediated NF‐κB‐dependent neuroinflammation and neuronal Aβ production by beta‐site APP cleaving enzyme 1. Furthermore, cannula‐delivered CatE inhibitors improved memory function and reduced Aβ accumulation and neuroinflammation in AD mice. Our findings reveal that CatE as a modulator of microglial activation and neurodegeneration in AD and suggest CatE as a therapeutic target for AD by targeting neuroinflammation and Aβ pathology.

## Graphical abstract

Microglial cathepsin E involves the secretion of TRAIL, resulting in microglia‐mediated neuroinflammation and neuronal Aβ production during AD. Cannula‐delivered Cathepsin E inhibitor improves memory function and reduced neuroinflammation and Aβ production in AD mice.

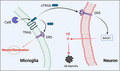

## 1 INTRODUCTION

Alzheimer's disease (AD) is a common neurodegenerative disorder characterized by accumulation of neuritic plaques, neurofibrillary tangles, and neuroinflammation in the brain, and is accompanied by cognitive impairment and memory loss (Long & Holtzman, 2019). Despite extensive basic research and clinical trials during the last four decades, there are no treatments that can prevent the progression of this disease. Genetic and epigenetic studies, transcriptome analysis of brains of patients with AD, and quantitative analysis of traits expressed in monocytes all support a role for the innate immune system in AD (Venegas et al., 2017). Microglia are resident immune cells of the central nervous system that are responsible for the maintenance of brain homeostasis (Marschallinger et al., 2020). Recent single‐cell transcriptomic studies have revealed several distinct subpopulations of microglia, including disease‐associate‐microglia, and cellular states during aging in neurodegenerative disease (Keren‐Shaul et al., 2017). In AD mouse models, the clearance of senescent glial cells has been shown to prevent cognitive decline (Bussian et al., 2018) and pharmacological elimination of microglia protected against neuronal loss and cognitive dysfunction (Spangenberg et al., 2019). These findings suggest a pivotal role for microglia in the progression of AD.

Cathepsins are a group of proteases in the endosomal‐lysosomal system. The primary function of cathepsins is to degrade proteins by bulk proteolysis in lysosomes. However, recent studies using gene knock‐out mice have revealed that cathepsins can carry out modulatory functions by limited proteolysis of proteins (Nakanishi, 2020). Cathepsin E (CatE), an aspartic protease, is barely detectable in normal healthy brain tissue but is highly expressed in activated microglia of pathological brains (Nakanishi et al., 1993; Ni et al., 2015; Sastradipura et al., 1998). CatE is upregulated and secreted from activated microglia, where it proteolytically liberates TNF‐related apoptosis‐inducing ligand (TRAIL) from the microglial surface and activates nuclear factor kappa B (NF‐κB), leading to chronic neuroinflammation and brain damage(Ni et al., 2015). We have reported that CatE‐deficient mice were resistant to mechanical allodynia in experimental autoimmune encephalomyelitis (EAE). Neutrophils produced CatE‐dependent elastase in dorsal root ganglia and were activated during the preclinical phase of EAE (Harada et al., 2019). In familial amyloidotic polyneuropathy (FAP), CatE was reported as a novel FAP biomarker and a possible modulator for innate immune cell chemotaxis to the disease affected tissues (Goncalves et al., 2017). Yet little is known about the role of CatE in AD, and CatE specific inhibitor is unavailable until grassystatin‐derived peptides were modified (Stotz et al., 2020).

To elucidate the function of CatE in AD pathogenesis, we crossed amyloid precursor protein (APP)‐knock‐in (KI) mice, an advanced AD mouse model called APP^NL−G−F^ mice that expresses human APP under the mouse endogenous APP promoter, with CatE‐deficient mice. Remarkably, CatE‐deficient AD mice exhibited amelioration of AD neuropathology, including cognitive deficits, Aβ plaques and neuroinflammatory responses. We have investigated the underlying mechanisms of these findings, and our results suggest that microglial CatE is required for soluble TRAIL (sTRAIL) liberation involved in microglia‐neuron communication, resulting in microglia‐mediated neuroinflammation and neuron‐mediated Aβ deposition.

## 2 RESULTS

### 2.1 CatE expression increases in the brains of AD mice and AD patients

**FIGURE 1:**
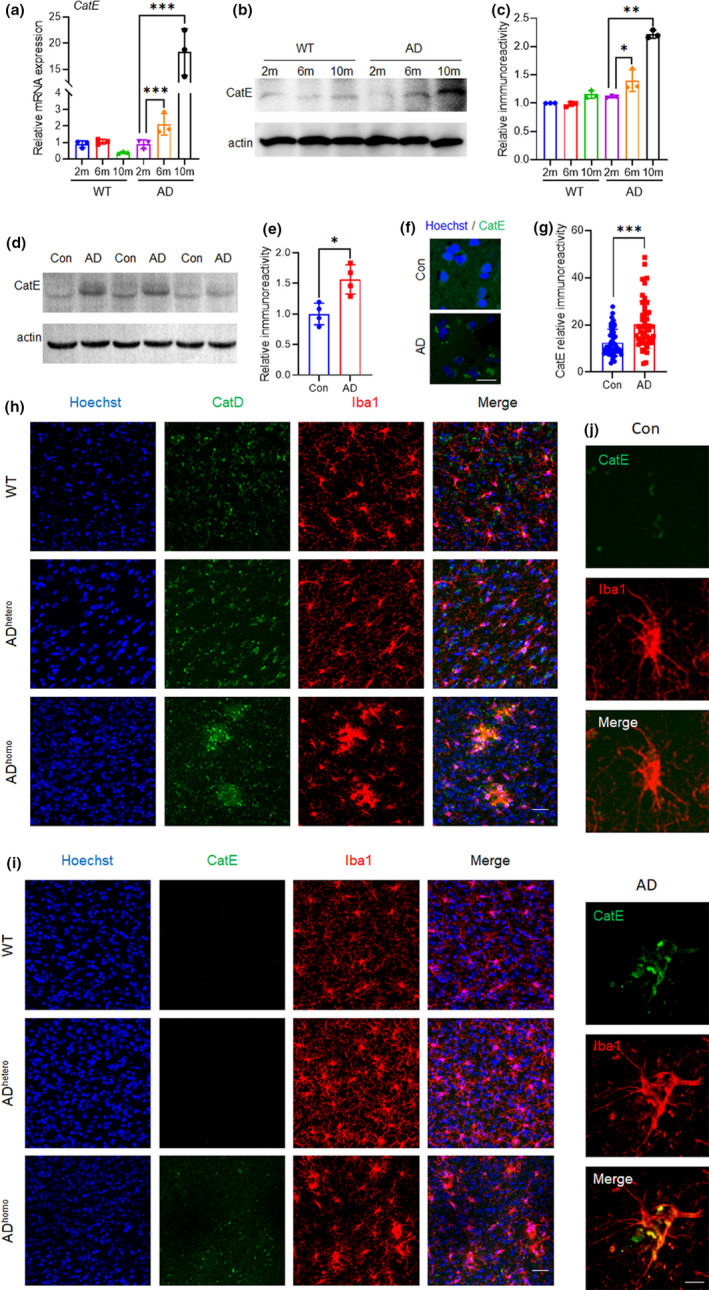
CatE levels increases in the microglia of Alzheimer's disease brains. (a), Relative mRNA expression of CatE in the hippocampus of WT and AD mice. Values are mean ± SEM (2‐, 6‐, and 10–month‐old, n = 3 mice/genotype). ***p < 0.001, One‐way ANOVA. (b), Immunoblot analysis of CatE in the hippocampus from WT and AD mice (2‐, 6‐, and 10‐month‐old). (c), Quantification of CatE in the immunoblots shown in (b). Values are mean ± SEM (2‐, 6‐, and 10‐month‐old, n = 3 mice/genotype). *p < 0.05, **p < 0.01, One‐way ANOVA. (d), Immunoblot analysis of CatE in the cortex from AD patients and the age‐matched control. (e), Quantification of CatE in the immunoblots shown in (d). Values are mean ± SEM (n = 4 cases in each group). **p < 0.01, Student's t‐test. (f), Immunofluorescent images of CatE in the cortex of AD patient and age‐matched control, Scale bar, 20μm. (g), Quantification of the fluorescent density of CatE shown in (f). 50 cells in each group were analyzed. Values are mean ± SEM (n = 3 cases/group). ***p < 0.001, Student's t‐test. (h, i) Immunofluorescent images of CatD and CatE in the cortex from 6‐month‐old WT, AD^hetero^, and AD^homo^ mice. Scale bar, 30 μm. (j), Immunofluorescent images of CatE (green) with Iba1 (red) in the cortex from AD patients and age‐matched control. Scale bar, 10 μm

To investigate the role of CatE in the pathology of AD, we initially examined the mRNA and protein expression levels of CatE in the cortex of AD mice ranging from 2‐ to 10‐ month‐old. CatE was barely detectable in the cortex of wild‐type (WT) mice at 2, 6 and 10 months of age (Figure 1a‐c), however, both mRNA and protein expression of CatE increased significantly in the cortex of AD mice at 6‐ and 10‐months of age compared with age‐matched WT mice (Figure 1a‐c). As was reported previously, essentially no CatE is detectable in healthy nervous tissue (Nakanishi et al., 1993, 1994), however, a significant increase in the protein was observed in rat hippocampus and striatum after transient forebrain ischemia (Nakanishi et al., 1993). In addition, CatE showed significantly increased and clustered expression in cortical tissue from a patient with AD compared with age‐matched controls (Figure 1f,g), which in consistence with the results obtained by immunoblotting (Figure 1d,e). CatE and CatD are representative lysosomal aspartic proteinases, the comparable expression between them were analyzed by immunostaining. Visible CatD were detected in WT, AD^hetero^, and AD^homo^ mice at 6‐month‐old (Figure 1h); however, CatE was rarely detectable in WT and AD^hetero^ mice but detectable in AD^homo^ mice at 6‐month‐old (Figure 1i). Next, the localization of CatE was examined to further elucidate its role in the pathology of AD. In the brains of 6‐month‐old AD mice, increased CatE immunoreactivity was observed in activated microglia but less detectable in astrocytes or neurons (Figure S1a). We further analyzed the expression pattern of CatE on brainranseq.org which is a user‐friendly website that provide a transcriptome from eight cell types (Y. Zhang et al., 2014). The result supported that microglia is the major CatE‐expressed cell type (Figure S1b). Staining of AD patient brains further supports the microglial localization of CatE (Figure 1k,j).

Aβ deposition is observed starting at two months and is nearly at its maximum by seven months in the brains of AD mice (Saito et al., 2014). Activated microglia were found surrounding the Thioflavin S^+^ and Aβ^+^ plaque in the cortex from 6‐month‐old AD mice (Figure S1c). Clustered expression of CatE in microglia (Figure S1c) prompted us to examine the relationship between CatE and Aβ deposition. These results suggest that microglial CatE may associate with Aβ metabolism.

### 2.2 Absence of CatE results in improved cognitive function and reduced plaque deposition in AD mice

**FIGURE 2:**
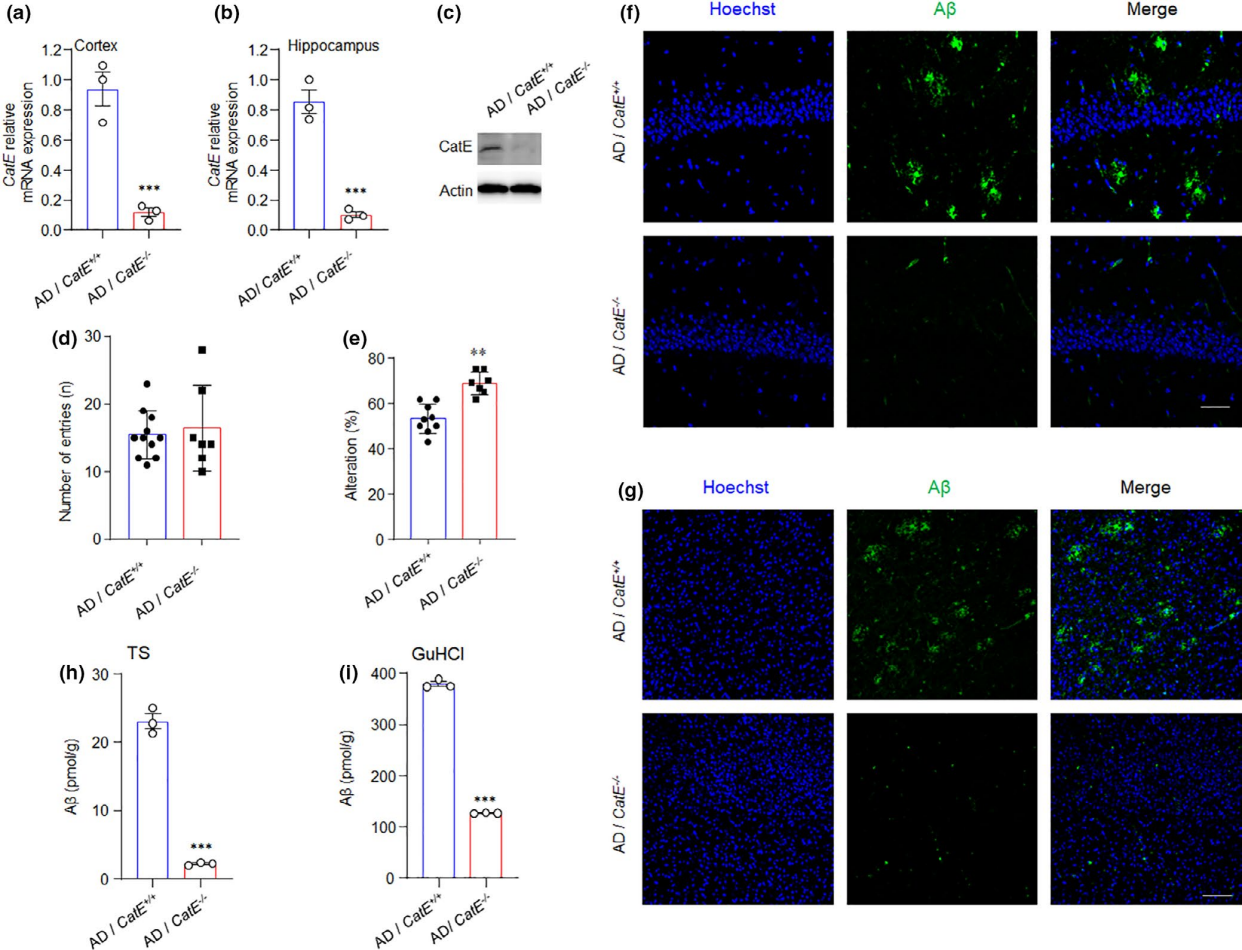
CatE deficiency ameliorates amyloidosis and memory decline in AD mice. (a, b), Relative mRNA expression of CatE in the cortex(a) and hippocampus (b) from 6‐month‐old AD mice and AD /*CatE* ^−/−^ mice. Values are mean ± SEM (n = 3 mice/genotype). ***p < 0.001, Student's *t*‐test. (c), Immunoblot analysis of CatE in the cortex from 6‐month‐old AD mice and AD /*CatE* ^−/−^ mice. (d) Spatial working and reference memory was evaluated by the Y‐maze test in 6‐month‐old AD mice and AD /*CatE* ^−/−^ mice. The total number of entries of each group (n = 7–11 mice/genotype). (e), The spontaneous alternation percentages for each group were calculated. The results in represent the mean ± SEM (n = 7–11 mice/genotype). **p < 0.01, Student's *t*‐test. (f, g), Fluorescent images of Aβ (green) with Hoechst (blue) in the hippocampus (f) and cortex (g) from 6‐month‐old AD and AD /*CatE* ^−/−^ mice. Scale bar, 200μm. (h, i), Concentration of Aβ in the Tris‐HCL‐buffered saline (TS) and GuHCL fractions of cortex from 6‐month‐old AD and AD /*CatE* ^−/−^ mice. Values are mean ± SEM (n = 3 mice/genotype). ***p < 0.001, Student's *t*‐test

It is now clear that lysosomal proteases can contribute to AD by proteolytic processing or via autophagy, which plays a major role in the disease through the clearance of accumulated material, including Aβ (Stoka et al., 2016). Thus, we hypothesized that the lack of CatE would result in higher Aβ plaque load because of reduced clearance activity of microglia, promoting AD pathologies. To test this hypothesis, we crossed mice deficient in CatE with AD mice. The double homozygous mice were examined for CatE mRNA and protein expression (Figure 2a‐c). Surprisingly, 6‐month‐old AD/*CatE* ^−/−^ mice showed a significantly higher percentage of alterations than did 6‐month‐old AD mice in a Y‐maze test, while no difference was observed between the two groups in the numbers of entries into each arm (Figure 2d,e). On the other hand, the amount of Aβ deposition was reduced in the cortex of 6‐month‐old AD mice lacking CatE (Figure 2f,g). Soluble and insoluble Aβ_1‐42_ were further examined by ELISA using cortical tissues dissolved in the TS and GuHCl fractions, respectively. Quantification of both soluble and insoluble Aβ_1‐42_ showed a statistically significant decrease in the cortex of 6‐month‐old AD/*CatE* ^−/−^ mice (Figure 2h,i). Our findings indicate that CatE deficiency reduces Aβ level and plaque deposition and rescues learning and memory deficits in 6‐month‐old AD mice.

### 2.3 Microglia lacking CatE have no effect on degradation of Aβ_1‐42_

In the brain, CatE is majorly expressed on microglia, allowing us to interpret changes in Aβ degradation activity of microglia with CatE deficiency. We found that CatE deficiency had no effect on the expression of tissue‐type plasminogen activator (tPA), insulin‐degrading enzyme (IDE) Aβ‐degrading enzymes including neprilysin (NEP) and angiotensin‐converting enzyme (ACE) in the cortex and hippocampus of 6‐month‐old AD and AD/*CatE* ^−/−^ mice (Figure S2a,b). Microglial cells secrete several enzymes capable of degrading extracellular Aβ (Hickman et al., 2008). We used an *in vitro* Aβ degradation assay system in which freshly solubilized Aβ_1‐42_ was added to *CatE* ^−/−^ and control microglial cells (cell‐based assay) or their cultured medium (cell‐free assay) for ELISA measurements (Czirr et al., 2017). The usage of Aβ_1‐42_ was evaluated by the cell viability assay using MG6 cells (Figure S3a) and 1 μM Aβ_1‐42_ in the following experiments. In both the cell‐based and cell‐free systems, there were no significant differences in phagocytic activity (Figure S2c and Figure S3b,c) and degradation of Aβ_1‐42_ (Figure S2c‐e and Figure S3d,e) between WT and *CatE* ^−/−^ primary microglia or CatE knock‐down MG6 cells. Notably, there were no obvious changes in the amount of Aβ_1‐42_ between WT and *CatE* ^−/−^ mice 5 days after stereotactic injection of Aβ_1‐42_ into the cortex (Figure S2f). These data indicate that the effects of CatE on Aβ_1‐42_ metabolism were not dependent on the phagocytotic and degradation activities of microglia.

Consistent with the results *in vivo*, the expression of CatE was very low in WT MG6 cells, but markedly increased after treatment with Aβ_1‐42_ (Figure S3d). Microglia express several receptors involved in the recognition, internalization, and clearance of Aβ and cell activation. We have previously reported that myelin oligodendrocyte glycoprotein (MOG_35‐55_)‐induced CatE upregulation is mediated through toll‐like receptor 4 (TLR4) in neutrophils (Harada et al., 2019). Therefore, we firstly addressed whether Aβ_1‐42_ activated microglia and upregulated CatE through TLR4. Lipopolysaccharide‐*Escherichia coli* (LPS), a TLR4 ligand, was used at different concentrations to stimulate MG6 cells for 48 h. An unexpected consequence of LPS stimulation was that although it did not induce the expression of CatE, homologous proteases including CatB and CatD, was upregulated in MG6 cells (Figure S4a,b). On the other hand, MG6 cells showed a Aβ_1‐42_ dose‐dependent increase in the expression of CatE, while CatB and CatD were highly expressed at 10 nM up to 1μM and 5 μM, respectively (Figure S4c,d). Therefore, these data showed that the regulation of CatE by Aβ and LPS in microglia is distinct from the regulation of CatB and CatD, which may attribute to different key regulative transcription factors. CatB and CatE may share the same regulation pattern in the presence of Aβ or LPS, but CatE not. *In vivo* results verified the augmented level of CatE in microglia of WT mice 5 d after stereotactic injection of Aβ_1‐42_ into the cortex (Figure S2g). The particular cell type expression of CatE may result from its regulation by cell‐specific transcription factors such as purine‐rich box‐1 (PU1) that are specifically expressed in microglia (Cook et al., 2001; Okamoto et al., 2012; Rustenhoven et al., 2018). As expected, DB1976, a PU1‐specific inhibitor, significantly reduced expression of CatE in MG6 cells after Aβ_1‐42_ treatment (Figure S2h). Further studies using promoter constructs of CatE to elucidate additional the Aβ‐regulated specific transcription factors in microglia will strengthen the present study.

### 2.4 Absence of CatE inhibits amyloidogenic processing of APP and Tau phosphorylation in AD mice

**FIGURE 3:**
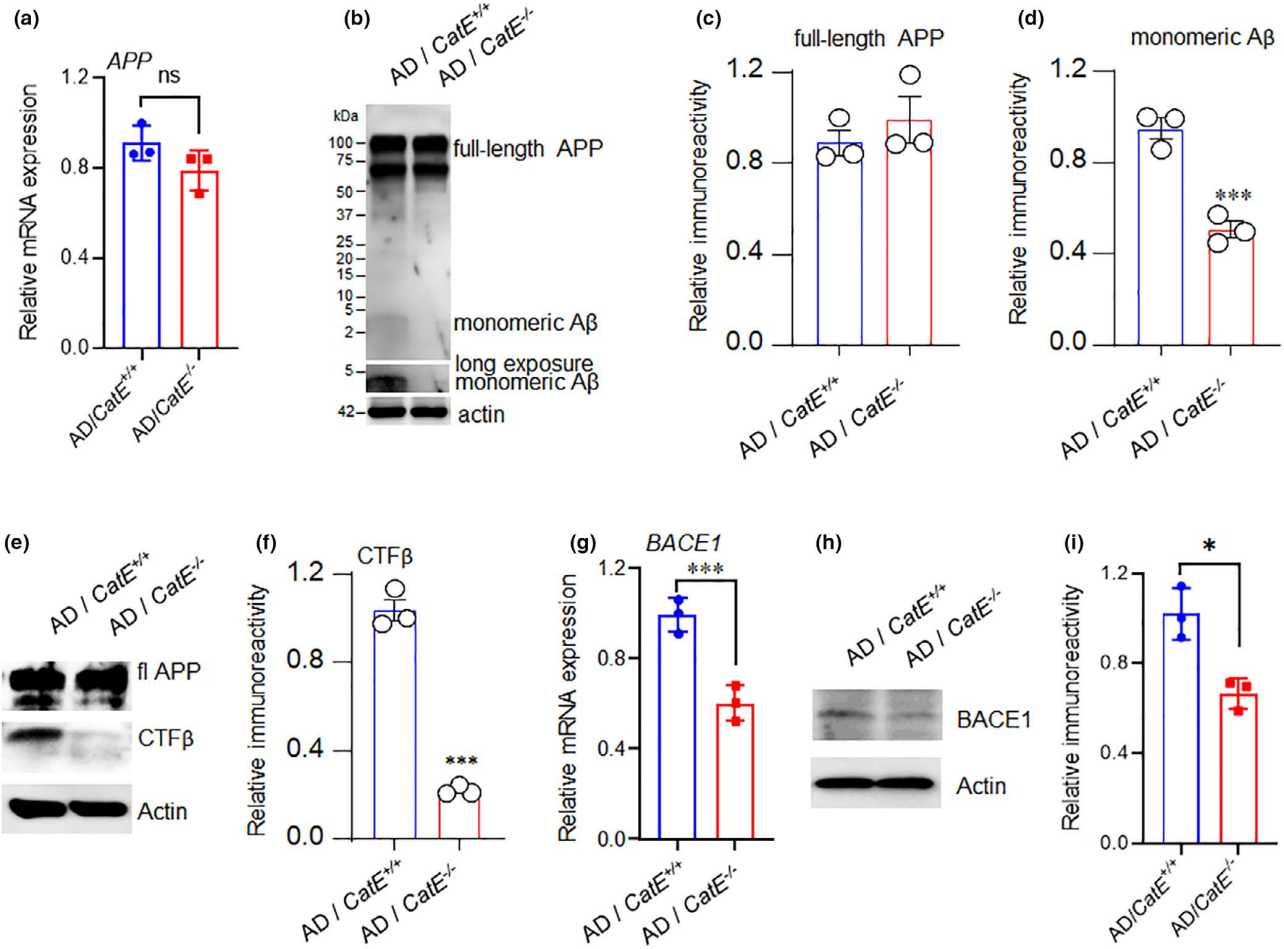
CatE deficiency ameliorates Aβ production in AD mice. (a), Relative mRNA expression of APP in the hippocampus of from 6‐month‐old AD and AD/*CatE* ^−/−^ mice. Values are mean ± SEM (n = 3 mice/genotype). (b), Immunoblot analysis of full‐length APP and Aβ in the hippocampus of from 6‐month‐old AD and AD/*CatE* ^−/−^ mice. (c, d), Quantification of APP (c) and Aβ (d) in the immunoblots shown in (b). Values are mean ± SEM (n = 3 mice/genotype). ***p < 0.001, Student's *t*‐test. (e), Immunoblot analysis of full‐length APP and CTFβ in the hippocampus of from 6‐month‐old AD and AD/*CatE* ^−/−^ mice. (f), Quantification of sAPPβ in the immunoblots shown in (e). Values are mean ± SEM (n = 3 mice/genotype). ***p < 0.001, Student's *t*‐test. (g), Relative mRNA expression of BACE1 in the hippocampus of from 6‐month‐old AD and AD /*CatE* ^−/−^ mice. Values are mean ± SEM (n = 3 mice/genotype). ***p < 0.001, Student's *t*‐test. (h), Immunoblot analysis of BACE1 in the hippocampus of from 6‐month‐old AD and AD/*CatE* ^−/−^ mice. (i), Quantification of BACE1 in the immunoblots shown in (h). Values are mean ± SEM (n = 3 mice/genotype). *p < 0.05, Student's *t*‐test

Next, we investigate whether a lack of CatE affects Aβ production in AD mice. AD mice were generated by knock‐in of a humanized Aβ sequence bearing AD‐associated mutations into the mouse APP locus under the control of endogenous promoter of mouse APP (Saito et al., 2014). CatE deficiency had no effects on APP mRNA and protein expression (Figure 3a‐c), which suggested that CatE deficiency had no effect on transcriptional regulation of APP. However, CatE deficiency significantly reduced the expression of Aβ and β‐cleavage products (CTFβ) and decreased β‐site APP cleaving enzyme 1 (BACE1) expression (Figure 3b, d, g‐i). These data suggested that decreased expression of BACE1 in CatE‐deficient microglial cells may account for the decreased deposition of Aβ *in vivo*. However, an early study found that CatE had major cleavage sites between residues 19/20 and 93/94 of βAPP100‐flag *in vitro*, which suggested it may have a role in the catabolism of both Aβ and APP (Mackay et al., 1997). CatE was recently classified into a group of proteases involved in Aβ degradation (Van Acker et al., 2021). Genetic data, as well as autopsy and neuroimaging research in patients with AD, have indicated that Aβ plaque deposition and cortical Tau pathology occurred simultaneously (Ciccone et al., 2020). In AD mice, Western blots further showed that phosphorylation of Tau protein at several residues, including 202 and 396, was significantly reduced by CatE deletion (Figure S5a‐d). These findings indicate that suppression of Tau‐hyperphosphorylation by CatE deficiency may be a result of its effect on Aβ. Additionally, CatE deficiency significantly increased postsynaptic density protein 95 (PSD95), an important synapse‐associated protein, in the cortex of AD mice (Figure S5e‐g). Together, these results suggested that CatE is responsible for enhanced Aβ production through BACE1 upregulation and may account for Tau pathology and synaptic dysfunction.

### 2.5 Absence of CatE attenuates inflammation in the brains of AD mice

**FIGURE 4:**
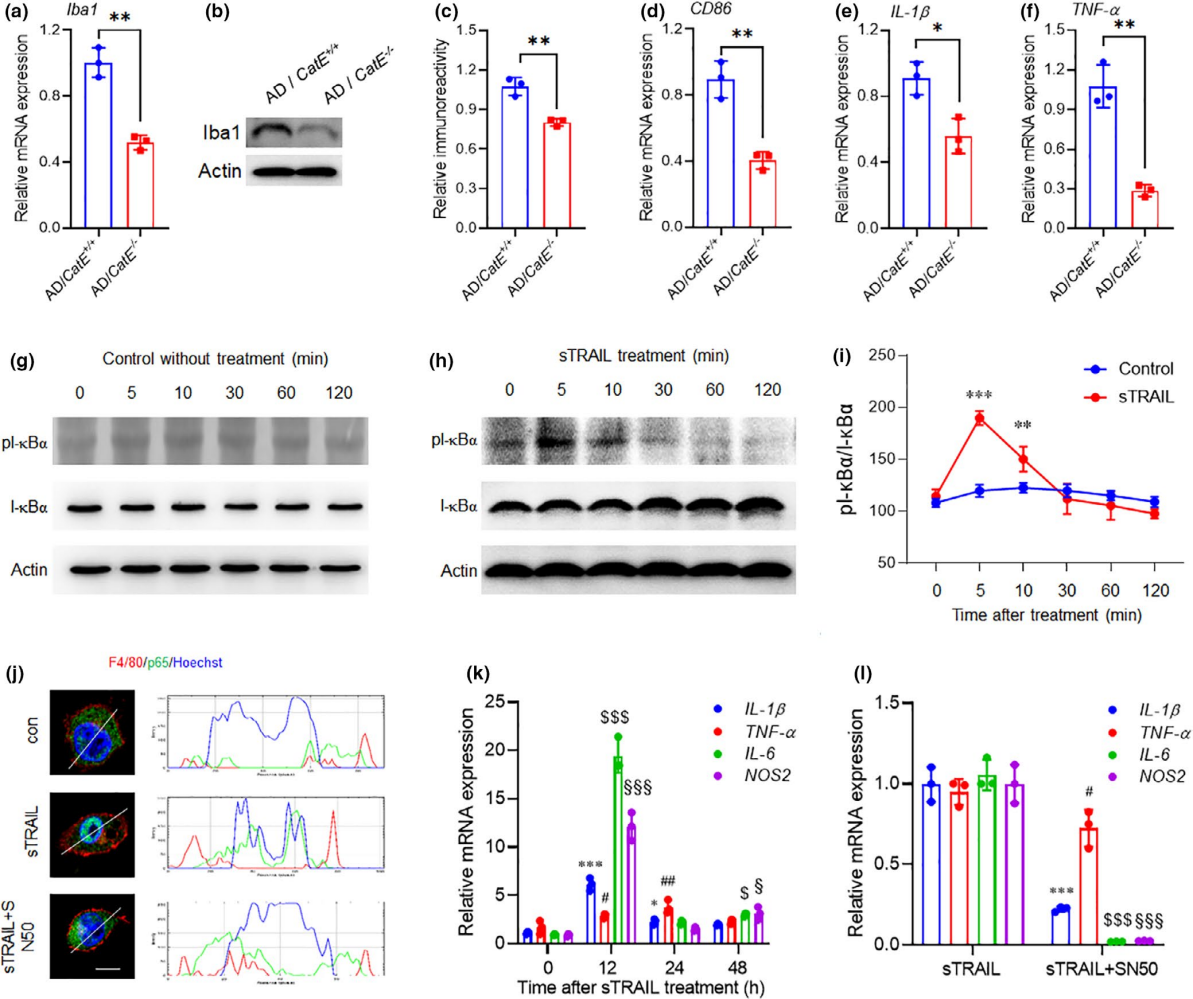
CatE deficiency ameliorates inflammatory responses in AD mice and MG6 cells. (a), Relative mRNA expression of Iba1 in the hippocampus of from 6‐month‐old AD and AD/*CatE* ^−/−^ mice. Values are mean ± SEM (n = 3 mice/genotype). **p < 0.01, Student's *t*‐test. (b), Immunoblot analysis of Iba1 in the hippocampus of from 6‐month‐old AD and AD/*CatE* ^−/−^ mice. (c), Quantification of Iba1 in the immunoblots shown in (b). Values are mean ± SEM (n = 3 mice/genotype). *p < 0.05, **p < 0.01, Student's *t*‐test. (d‐f), Relative mRNA expression of CD86, IL‐1β, and TNF‐α in the hippocampus of from 6‐month‐old AD and AD/*CatE* ^−/−^ mice. Values are mean ± SEM (n = 3 mice/genotype). *p < 0.05, **p < 0.01, Student's *t*‐test. (g, h), Immunoblot analysis of pI‐κBα and I‐κBα in the MG6 microglia without (g) or with (h) 100ng/ml sTRAIL treatment for 0, 5, 10, 30, 60, and 120min. (i), Quantification of pI‐κBα/I‐κBα in the immunoblots shown in (g, h). Values are mean ± SEM (n = 3 culture preparations). **p < 0.01, ***p < 0.001, Two‐way ANOVA. (j), Immunofluorescent images of p65 (green) with F4/80 (red) and Hoechst (blue) in the MG6 microglia cells after 100ng/ml sTRAIL treatment for 5 min. SN50 was applied 2h prior to sTRAIL and treated as a negative control. Scale bar, 5μm. The right profile plots fluorescence intensity (values from 0 to 250) of p65 (green), F4/80 (red), and Hoechst (blue) at the position along the white lines in the corresponding left panels, respectively. (k), Relative mRNA expression of IL‐1β, TNF‐α, IL‐6, and NOS2 in MG6 microglia cells after 100ng/ml sTRAIL treatment for 0, 12, 24, and 48h. Values are mean ± SEM (n = 3 cell culture preparations). *p < 0.05, ***p < 0.001, ^#^ *p *< 0.05, ^##^ *p *< 0.01, ^$^ *p *< 0.05, ^$$$^ *p *< 0.001, ^§^ *p *< 0.05, ^§§§^ *p *< 0.001, One‐way ANOVA. (l), Relative mRNA expression of IL‐1β, TNF‐α, IL‐6, and NOS2 in MG6 microglia cells after 100ng/ml sTRAIL treatment for 12 h in the presence or absence of SN50. Values are mean ± SEM (n = 3 cell culture preparations). ***p < 0.001, ^#^ *p *< 0.05, ^$^ *p *< 0.05, ^$$$^ *p *< 0.001, and ^§§§^ *p *< 0.001, One‐way ANOVA

Microglia make up the innate immune system of the central nervous system and are key cellular mediators of neuroinflammatory processes (Ni et al., 2019). Therefore, the effects of CatE deficiency on neuroinflammation in microglia were assessed. CatE deficiency significantly reduced both mRNA and protein expression of ionized calcium‐binding adaptor protein (Iba1), a pan‐microglial marker whose expression increases with microglial activation (Figure 4a‐c). Next, typical pro‐inflammatory mediators, including cluster of differentiation 86 (CD86), interleukin‐1β (IL‐1β), and tumor necrosis factor‐α (TNF‐α), were examined by RT‐PCR, CatE deficiency significantly reduced the expression of CD86, IL‐1β, and TNF‐α mRNA in the cortex of AD mice (Figure 4d‐f). These results indicated that CatE may regulate neuroinflammation in microglia, which agrees with previously published data that CatE/CatB cooperate to activate NF‐κB and promote chronic neuroinflammation in a hypoxia‐ischemic mouse model (Ni et al., 2015).

CatE has been reported to be involved in the proteolytic activation of TRAIL in microglia, and blockade of sTRAIL by neutralizing antibodies has been shown to result in functional improvement and a restrained immune/inflammatory response in the brains of 3xTg‐AD mice *in vivo*(Cantarella et al., 2015; Ni et al., 2015). Building on these previous studies, we hypothesized that microglial CatE contributes to the secretion of sTRAIL, which binds to the receptors on both microglia and neurons. To explore this possibility, we used the MACS method to separately isolate microglia and neurons from 6‐month‐old WT and AD mice. RT‐PCR data showed that TRAIL mRNA levels increased in microglia of AD mice compared with those in WT mice (Figure S6a). We found that the expression of the sTRAIL receptors DR5, mDctrailR1, and OPG was unchanged, while mDctrailR2 levels increased in microglia of AD mice compared with WT mice (Figure S6b‐e), however, all four receptors showed increased mRNA expression in neurons of AD mice compared with WT mice (Figure S6f‐i). Analysis of AD patients’ samples also showed an increase in TRAIL mRNA and sTRAIL protein in AD (Figure S6l, m), along with an increase in sTRAIL receptor levels (Figure S6n‐r). In addition to the examination of the distribution of TRAIL in microglia and its receptors on both microglia and neurons, we also evaluated the role of CatE in the secretion of sTRAIL in microglia. CatE deficiency significantly reduced expression of TRAIL mRNA in AD mice (Figure S7a), suggesting that CatE may contribute to the transcription of TRAIL. CatE overexpression vectors markedly increased expression of CatE in MG6 cells (Figure S7f, g), and significantly increased secretion of sTRAIL into the culture medium (Figure S7h). Interestingly, a combination of CatE overexpression and Aβ treatment in MG6 cells induced higher secretion of sTRAIL compared with overexpression of only CatE (Figure S7h), which suggested CatE may have a greater effect on sTRAIL secretion in AD brains. In addition, treatment of MG6 cells with human recombinant CatE (hCatE) also significantly increased sTRAIL levels compared with control (Figure S7h), possibly indicating working pH of CatE is near neutrality.

We treated MG6 cells with sTRAIL to explore its possible involvement in the activation of NF‐κB and subsequent neuroinflammatory responses. Phosphorylated IκBα was peaked in MG6 cells at 5 min after treatment with sTRAIL, but showed no change in the control (Figure 4g‐i), indicating marked activation of NF‐κB by sTRAIL. Activated NF‐κB translocates into the nucleus and promotes gene transcription for downstream events. To further confirm the activation of NF‐κB, we traced the localization of p65, a subunit of NF‐κB, in MG6 cells with or without sTRAIL treatment in the presence of SN50, an inhibitor of NF‐κB translocation. Immunofluorescent staining showed an obvious nuclear translocation of p65 in MG6 cells after treatment with sTRAIL, which was diminished by pretreatment with SN50 (Figure 4j). In addition, RT‐PCR showed a chronic inflammatory response in MG6 cells 12 to 48 h after sTRAIL treatment, which peaked at 12 h (Figure 4k). As expected, SN50 pretreatment significantly inhibited mRNA expression of pro‐inflammatory mediators (Figure 4l).

Collectively, our findings indicate that the CatE/TRAIL axis is responsible for the activation of NF‐κB and subsequent inflammatory responses in microglia.

### 2.6 sTRAIL contributes to amyloidogenic processing of APP in neurons

**FIGURE 5:**
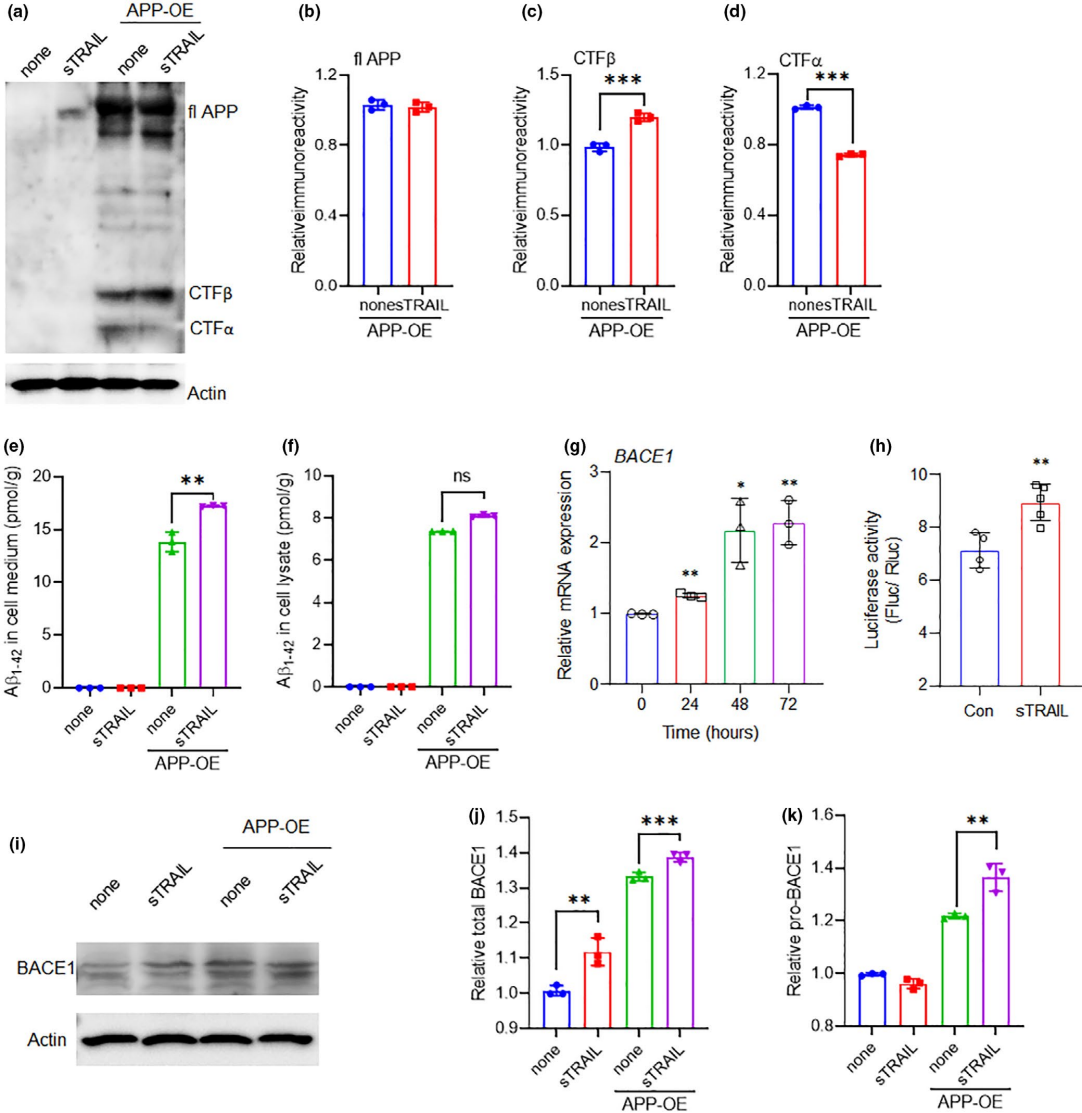
sTRAIL induces Aβ production in neurons. (a), Immunoblot analysis of full‐length APP, sAPPβ, CTFβ, and CTFα in N2a cells after 48h treatment with 100ng/ml sTRAIL or combination with APP overexpression for 72 h. (b‐e), Quantification of APP (b), CTFβ (c), and CTFα (d) in the immunoblots shown in (a). Values are mean ± SEM (n = 3 cell culture preparations). ***p < 0.001, Student's *t*‐test. (e, f), Amount of Aβ in the cell lysates (e) and cell culture medium (f) in N2a cells after 48h treatment with 100ng/ml sTRAIL or combination with APP overexpression for 72 h. Values are mean ± SEM (n = 3 cell culture preparations). **p < 0.01, Student's *t*‐test. (g), Relative mRNA expression of CatE in MG6 microglia cells after 100ng/ml sTRAIL treatment. Values are mean ± SEM (n = 3 cell culture preparations). *p < 0.05, **p < 0.01, ***p < 0.001, One‐way ANOVA. (h), Luciferase assay of BACE1 promoter in MG6 cells after 100ng/ml sTRAIL treatment for 48 h. Values are mean ± SEM (n = 3 cell culture preparations). **p < 0.01, Student's t‐test. (i), Immunoblot analysis of BACE1 in N2a cells after 48h treatment with 100ng/ml sTRAIL or combination with APP overexpression for 72 h. (j), Quantification of total BACE1 in the immunoblots shown in (i). Values are mean ± SEM (n = 3 cell culture preparations). **p < 0.01, ***p < 0.001, One‐way ANOVA. (k), Quantification of pro‐BACE1 in the immunoblots shown in (i). Values are mean ± SEM (n = 3 cell culture preparations). **p < 0.01, One‐way ANOVA

In *in vitro* studies, we overexpressed human APP containing Swedish mutations by transfecting a N2a neuronal cell line in the absence or presence of sTRAIL treatment. Full‐length APP was observed in the transfected N2a cells, while no corresponding bands were detected in untransfected cells (Figure 5a). sTRAIL had no effect on APP expression in the transfected N2a cells (Figure 5a, b), however, it induced significantly increased level of CTFβ (Figure 5a, c), and decreased CTFα (Figure 5a, d). Increased β‐secretase cleavages prompted us to examine the production and secretion of Aβ in the cultured N2a cells. We found sTRAIL significantly increased the secretion of Aβ into the culture medium, but not in cell lysate from N2a cells with APP‐OE (Figure 5 e, f). In addition, sTRAIL significantly increased BACE1 mRNA expression and promoter activity of in N2a cells (Figure 5g, h). Furthermore, sTRAIL induced significantly increased expression of total and pro‐type BACE1 in N2a cells with and without APP‐OE (Figure 5i‐k). It was observed that sTRAIL induced increased pro‐BACE1 in N2a cells with APP‐OE consistent with the results in AD mice.

### 2.7 Targeting CatE with specific peptides results in reduced Aβ accumulation and neuroinflammation

**FIGURE 6:**
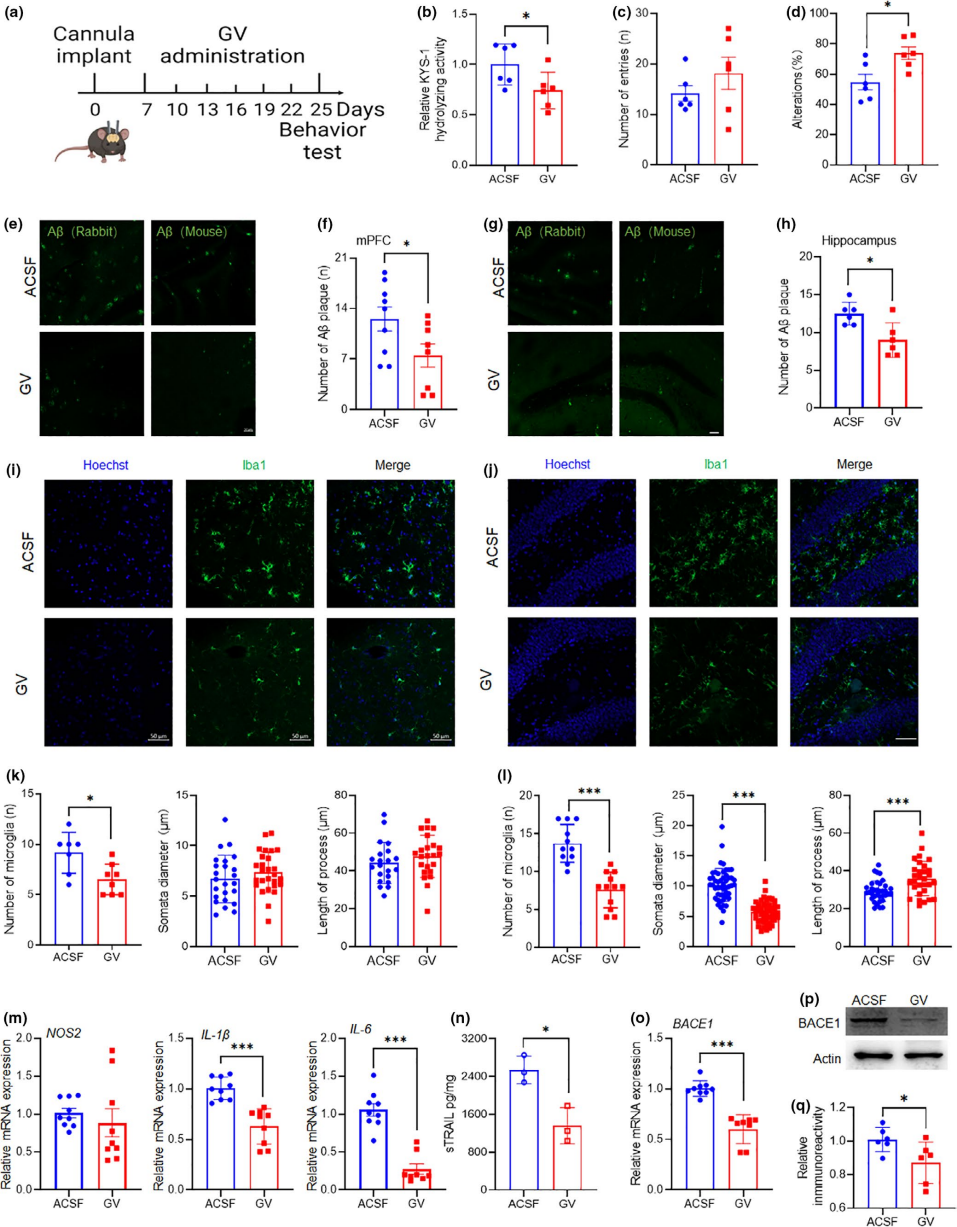
GV administration ameliorates inflammation and Aβ production in AD mice. (a), Experimental timeline. (b), CatE enzymatic activity assay in the cortical lysates from AD mice with GV or ACSF administration. Values are mean ± SEM (n = 6 mice/genotype). *p < 0.05, Student's *t*‐test. (c, d), Spatial working and reference memory were evaluated by the Y‐maze test in AD mice after GV or ACSF administration. The total number of entries of each group (c) and the spontaneous alteration percentages for the two groups (d) were calculated. The columns and bars represent the mean ± SEM (n = 6 mice in each group). *p < 0.05, Student's *t*‐test. (e, g), Immunofluorescent images of Aβ (green) in the cortex (e) and hippocampus (g) from AD mice after GV or ACSF administration. Scale bar, 50μm. (f, h), Quantification of amount of Aβ plaque shown in (e and f). Values are mean ± SEM (n = 5–6 mice in each group). *p < 0.05, Student's *t*‐test. (i, j), Immunofluorescent images of Iba1 (green) and Hoechst (blue) in the cortex (i) and hippocampus (j) from AD mice after GV or ACSF administration. Scale bar, 50μm. (k, l), Quantification of the microglial cell number, soma diameter and length of process in the cortex (k) and hippocampus (l) from AD mice after GV or ACSF administration. Values are mean ± SEM (n = 5–6 mice in each group). *p < 0.05, ***p < 0.001, Student's *t*‐test. (m), Relative mRNA of *NOS2*, *IL*‐*1*β, and *IL*‐*6* in the cortex from AD mice after GV or ACSF administration. Values are mean ± SEM (n = 3 mice in each group). ***p < 0.001, Student's *t*‐test. (n), The amount of sTRAIL in the cortex from AD mice after GV or ACSF administration. Values are mean ± SEM (n = 3 mice in each group). *p < 0.05, Student's *t*‐test. (o‐q), Relative mRNA(o) and protein (p, q) expression of BACE1 in the cortex from AD mice after GV or ACSF administration. Values are mean ± SEM (n = 3–4 mice in each group). *p < 0.05, Student's *t*‐test

Our data suggested that CatE is critical in neuroinflammatory responses and Aβ production. Specific inhibition of CatE without affecting highly abundant CatD was only possible using a protease inhibitor protein from *Ascaris suum*, but grassystatin peptides from marine cyanobacteria were more recently identified as potent inhibitors of this enzyme (Kwan et al., 2009). Simplified grassystatin variants (GV) have been reported to selectively inhibit CatE, and there is some indication of inhibition of CatD and napsin A (NapsA) (Stotz et al., 2020). The failure of therapies based on administration of peptides via intravenous or oral routes is often due to their inability to cross the blood‐brain barrier (BBB), therefore, we chose direct cannulation for delivery of GV into mouse brains (Figure 6a). Inhibition of CatE by GV was assessed in mouse brain tissue lysates using a specific fluorescence‐quenching substrate. GV administration significantly inhibited the enzymatic activity of CatE in the brain lysates of AD mice (Figure 6b). Y‐maze tests showed a significant increase in spontaneous alteration, but not total entries in AD mice after GV administration (Figure 6c, d). Consistent with our hypothesis, two antibodies with different epitopes used as probes showed that GV administration significantly reduced the amount of Aβ plaque in the cortex and hippocampus of AD mice compared with control groups administered artificial cerebrospinal fluid (ACSF) (Figure 6e‐h). Next, we assessed changes in inflammatory responses in the brains of these mice. Confocal laser scanning microscopy of Iba1 revealed a significant reduction in microglial cell numbers in both the cortex and hippocampus (Figure 6i‐l), and significant elongation of microglial processes and shrinking of their cell bodies were observed in the hippocampus (Figure 6j, l), which indicated that GV reduced microglial activation by inhibiting CatE. Furthermore, levels of NOS2, IL‐1β, and IL‐6 mRNA significantly decreased after GV administration (Figure 6m), as did the amount of sTRAIL and expression of BACE1 (Figure 6n‐q). Collectively, these data suggest that the inhibition of CatE with GV improves cognitive function by reducing neuroinflammation and Aβ production.

## 3 DISCUSSION

The present study revealed that microglial CatE is upregulated in the AD brain and has effects on both microglia and neurons that are dependent on sTRAIL secretion. Our results also indicate that the CatE‐sTRAIL axis is an inducer of neuroinflammation as it plays a role in modulation of microglial activation through NF‐κB, and is also a contributor to Aβ formation since it upregulates the expression of BACE1, which is a key protease involved in Aβ formation (Figure S8). Therapeutics targeting only one of AD‐related subpathologies have not been successful in the search for a disease‐modifying treatment. Therefore, a challenge in AD therapy is the identification of pharmaceutical targets that simultaneously affect several subpathologies. Pharmacological inhibition of CatE by GV improved cognitive function of AD mice as a consequence of interfering with neuroinflammation and Aβ production. Our study has major implications as it shows that CatE promotes AD progression through microglial neuroinflammation and neuronal Aβ production, which suggests potential targets for therapeutic interventions against AD. Such insight can help to guide future design of inflammation‐related diseases treatments.

We found that CatE was expressed at very low levels in the brains of WT mice and healthy human patients, but significantly upregulated in progression of AD, suggesting a pivotal role for CatE in that pathology. CatE has been found in the endosomal structures of microglia (Sastradipura et al., 1998), which is in consistent with our immunostaining results in the brain of AD mice and human patients. In contrast, CatE was also reported to be expressed in most large cortical and hippocampal pyramids and in neuron of the Nuc. basalis of Meynert (Bernstein et al., 1994). The discrepant finding may result from the phagocytosis of degenerating neurons by microglia or the different antibodies. The particular cell type expression of CatE may result from its regulation by cell‐specific transcription factors such as PU1 that is specifically expressed in microglia (Cook et al., 2001; Okamoto et al., 2012; Rustenhoven et al., 2018). Our *in vitro* cell culture results demonstrated that the inhibition of PU1 reduced CatE mRNA expression in the presence of Aβ, suggesting PU1 regulated CatE expression during progression of AD. On the contrary, it has been reported that Kaiso binds to the mCGCG motif in the Kaiso regulatory region and represses CatE transcriptional activity (Hiramatsu et al., 2019). Therefore, it would be interesting to examine the repressive activity of Kaiso on CatE before and during onset of AD.

CatE catalyzes the proteolytic release of sTRAIL from the cell surface (Kawakubo et al., 2007; Ni et al., 2015), which can induce the activation of NFκB through TRAIL receptors (Hu et al., 1999; L. Zhang et al., 2015). In the present study, we found that increased CatE was involved in the production of sTRAIL in microglia in the presence of Aβ. Notably, the expression of TRAIL was significantly increased in microglia purified from the brains of 6‐month‐old AD mice, and death receptor 5 (DR5), the major receptor of sTRAIL, was upregulated in both microglia and neurons from the brains of 6‐month‐old AD mice. These results suggested that CatE‐TRAIL‐mediated sTRAIL could have effects on both microglia and neurons. In fact, sTRAIL immunoneutralization by means of a monoclonal antibody is associated with a significant rescue of neurons from death (Cantarella et al., 2003), reduced accumulation of Aβ, and attenuated expression of inflammatory/immune mediators (Cantarella et al., 2015), and is paralleled by re‐balancing of both central and peripheral immune responses (Di Benedetto et al., 2019). Taking these observations together with the present results, we conclude that CatE‐TRAIL‐mediated sTRAIL may be involved in cross‐talk between microglia and neurons in progression of AD.

Microglial activation is thought to be a double‐edged sword in AD, as activated microglia can phagocytose Aβ and tau and prevent proteopathy, whereas excessive inflammatory activation can accelerate plaque accumulation and synapse loss (Lananna et al., 2020). Previous studies have found several molecules that can anti‐inflammatory and /or neuroprotective in the setting of AD, including 5‐lipoxygenase, CatB, and receptors including class A scavenger receptors and toll‐like receptors (Heneka et al., 2015; Wu et al., 2017; Yan et al., 2021), almost all of which have normal physiological functions and whose inhibition may cause side effects. Silent physiological and activated pathological functions of CatE indicate its potential use in therapeutic interventions. In cultured MG6 cells, sTRAIL induced a NFκB‐dependent pro‐inflammatory response. We have previously reported that CatE is regulated by NFκB in MG6 cells deprived of oxygen and glucose (Ni et al., 2015). Therefore, persistent activation of NFκB may further enhance the CatE‐TRAIL axis, resulting in chronic neuroinflammation in the AD brain.

Although a number of AD mouse models have been developed based on APP overexpression, this may yield additional phenotypes unrelated to AD, including the overproduction of soluble N‐terminal fragments, C‐terminal fragment‐α, C‐terminal fragment‐β, and APP intracellular fragments (Sasaguri et al., 2017). To overcome these drawbacks, a novel AD mouse model (single humanized APP‐KI mice carrying Swedish (NL), Beyreuther/ Iberian (F), and Arctic (G) mutations was generated by KI of a humanized Aβ sequence bearing AD‐associated mutations to the mouse APP locus (Saito et al., 2014). The AD mice generated equal amounts of APP as WT mice (Saito et al., 2014), therefore, the APP‐KI mice may show relatively more late‐onset AD (LOAD) phenotypes compared with other transgenic AD mouse models. However, neuronal cell death and neurofibril tangle occurred in LOAD were not occur in APP‐KI mice, which was not studied here. In the present study, we found that CatE deficiency in APP‐KI mice had no effect on the expression of APP. Interestingly, the amounts of Aβ, sAPPβ, and CTFβ were reduced in AD mice with the CatE deletion, which suggested CatE may enhance APP processing at the β‐cleavage site. As expected, CatE deficiency significantly reduced the expression of BACE1. Furthermore, *in vitro* N2a cell culture results suggested that sTRAIL induces increased β‐cleavage through increased BACE1. A BACE1 promoter activity assay also confirmed transcriptional regulation of BACE1 by sTRAIL. It has been reported that the BACE1 promoter harbors functional binding sites for numerous transcription factors including specificity protein 1, Yin Yang 1, peroxisome proliferator‐activated receptor γ, NFκB, hypoxia‐inducible factor 1, and signal transducer and activator of transcription 3 (Chami & Checler, 2012). However, we did not further identify the key transcription factors in the linkage between sTRAIL‐DR5 and the BACE1 promoter. Increased Aβ can further activate BACE1 to form a positive regulatory loop that sets off a vicious cycle, resulting in the accumulation of Aβ.

CatE, CatD, and NapsA share a similar substrate and inhibitor profile, rendering selective inhibition of a single enzyme for functional analysis difficult. The most obvious role of CatD in AD is its involvement in the clearance of Aβ and tau protein through the autophagy‐lysosomal system (Vidoni et al., 2016), while the role of NapsA in AD is unknown. Accordingly, peptides that specifically inhibit CatE appear to be particularly important in clarifying its functions. Stotz et al., modified grassystatin‐derived peptides that provided a useful approach for us to interfere with CatE enzymatic activity in the brains of AD mice (Stotz et al., 2020). In our experiments, cannulation‐delivered GV elicited functional improvement in AD mice, which exhibited increased cognitive function, reduced neuroinflammation, and reduced Aβ accumulation compared with the ACSF group. However, we have not addressed the exact location of CatE in terms of activation of TRAIL. On the contrary, there is a difference between hippocampus and cortex in the effectiveness of GV in altering microglia phenotype, which may attributed to the location of cannula. Recently, a single molecule array platform was developed to detect CatB in serum or plasma that offers a 1000‐fold increase in sensitivity and vastly reduced variance compared with colorimetric tests (Thangavelu & Boutte, 2021). Therefore, the small changes above endogenous levels of CatE in the CSF and serum of AD patients can now be studied.

In the present study, we used undifferentiated N2a neuroblastoma, MG6 microglial cells, and primary cortical neurons to study molecular events associated with Aβ phagocytosis, production, and related inflammatory responses. While these models have been extensively used for shaping our current knowledge of neurodegeneration in AD, the chronic nature of neurodegeneration cannot be appropriately examined under these *in vitro* conditions and must be complemented by animal models and human tissue. Accordingly, we obtained results from the APP‐KI mouse model of AD that exhibits endogenous expression of APP and cognitive deficits and showed their relevance to human brain samples from AD patients and age‐matched controls. However, it is necessary to bear in mind the inevitable limitations inherent in human samples, including variation in procedures for post‐mortem tissue retrieval.

## 4 CONCLUSION

In summary, both the lack of CatE and treatment with the CatE‐inhibiting GV lead to reductions in both neuroinflammation and Aβ accumulation *in vivo*. Furthermore, there were increases in both CatE and proteolytically liberated sTRAIL in microglia in the presence of Aβ. sTRAIL induced both NF‐κB‐dependent inflammatory responses in microglia and BACE1‐mediated‐Aβ production in neurons. To date, little has been known about the effects of CatE on the pathology of AD, and specific inhibitor of CatE was unavailable until the discovery of GV. We provide the first evidence that the CatE‐sTRAIL axis contributes to cross‐talk between microglia and neurons in the progression of AD, and that targeting CatE with GV resulted in significant functional recovery in AD mice. Therefore, CatE may be a potential therapeutic target for the treatment or prevention of AD.

## 5 EXPERIMENTAL PROCEDURES

### 5.1 Animals

WT, CatE knock‐out, and APP‐KI mice (AD mice) of C57BL/6 background were kept and bred in the Animal room of Beijing Institute of Technology and Kyushu University. The *CatE* ^−/−^ mice were obtained by crossing male and female *CatE* ^+/−^mice (Ni et al., 2015). The AD mice were kindly provided by Dr. Saido, Riken Brain Science Institute, Japan. The line of APP‐KI mice carried the Arctic mutation, Swedish, and Beyreuther/Iberian mutations. The selection of APP‐KI homozygous mice from their littermates obtained by heterozygous coupling was performed according to previously reported (Saito et al., 2014). *CatE* ^−/−^ mice were crossed to AD mice to obtain the AD/*CatE* ^−/−^ and AD/*CatE* ^+/−^ mice. The primers for genotyping were list in Table S1. Matched number of mice in both genders was used in the study. All animal studies were carried out in accordance with the guidelines contained in Regulation of Laboratory Animals (Beijing Institute of Technology and Kyushu University) and under the protocols approved by the Institutional Animal Care and Use committee review panels at Beijing Institute of Technology.

### 5.2 Case selection

Post‐mortem frontal cortex tissues from 4 control cases (84/F, 81/F, 83/M, 92 M) and 4 late‐onset AD (86/F, 82/F,82/M, 87/M) cases at Braak 4–6 stages were select from the National Health and Disease Human Brain Tissue Resource Center. The cases were matched as closely as possible in terms of the age at death, gender, and post‐mortem interval. All patients had signed an informed consent and agreed to the use of their brain material for medical research. In this study, the principles of the Declaration of Helsinki regarding the use of human brain samples were strictly observed. The use of the study samples was approved by the ethics committee of Beijing Institute of Technology.

### 5.3 Tissue collection and sample preparation

Mice were anesthetized with somnopentyl (50 mg/kg, Kyoritsu Seiyaku) and perfused with ice‐cold PBS. The brain was bisected for biochemical analysis and histological analysis. The cortices and hippocampi were carefully dissected out under a dissection microscope. Tissues for biochemical analysis were stored in −80℃ before further processing and tissues for histological analysis were fixed in 4% PFA overnight followed by submergence in 30% sucrose before freezing.

### 5.4 Cell isolation

Six‐month‐old WT and AD mouse brains were collected and enzymatically digested using the Neural Tissue Dissociation Kit (Miltenyi Biotec) at 37°C. Further processing was performed at 4°C. Tissue debris were removed by passing the cell suspension through a 30μm cell strainer and magnetically labeled with CD11b microbeads, the cells were extensively washed and separated in a magnetic field using MS columns (Miltenyi Biotec), and the CD11b‐positive fraction was collected. CD11b‐negative fraction was collected for further neuron purification using Neuron Isolation Kit, mouse (Miltenyi Biotec).

### 5.5 Immunoblotting analyses

Human and mouse brain and cultured cells were homogenized in RIPA buffer, and the samples in equal amount of protein were loaded on 7.5, 12, and 15% SDS‐polyacrylamide gels and then transferred to 0.4μm PVDF membranes. Specially, the level of soluble Aβ was analyzed using tricine‐SDS‐PAGE and transferred to 0.2μm PVDF membranes according to the published methods (Schagger, 2006). The blots were probed with the following antibodies: goat anti‐CatE (1:1000; R&D; AF1130); goat anti‐CatE (1:1000; R&D, AF1294); mouse anti‐CatD (1:1000; Santa Cruz Biotechnology, sc‐377299); mouse anti‐CatB (1:1000; Santa Cruz Biotechnology, sc‐365558); mouse anti‐Aβ (1:1000; Covance; SIG‐39320); rabbit anti‐APP C‐Terminal (1:1000; Millipore; 171610); mouse anti BACE1 (1:1000; Millipore; MAB5308); goat anti IL‐1β (1:1000; R&D; AF‐401‐NA); rabbit anti‐Iba1 (1:1000; Wako019‐19741); rabbit anti‐post synaptic protein 95 (PSD95) (1:2000; abcam; ab18258); rabbit anti‐synaptophysin (SYP) (1:2000; abcam; ab14692); rabbit anti‐S202 (1:5000; abcam; ab108387); rabbit anti‐S396 (1:5000; abcam; ab109390); anti‐T231(1:5000; abcam; ab151559); mouse anti‐Tau5 (1:5000; abcam; ab80579); rabbit anti‐IκBα (1:1000; Cell Signaling Technology; Cat# 8993); mouse anti‐pIκBα (1:1000; Cell Signaling Technology; Cat# 9246); and mouse anti‐actin (1:5000; abcam; ab49900). Primary antibodies were incubated overnight at 4°C and followed by incubation with second antibodies for 1h at room temperature before washing by TBST for 3 times. The HRP‐labeled antibodies were detected by an ECL kit with image analyzer (LAS‐41000; Fuji Photo Film).

### 5.6 Immunofluorescent staining

Brain sections and fixed cells were blocked in the blocking buffer (3% BSA, and 0.3% Triton X‐100 in PBS) for 1h at room temperature and then incubated with primary antibodies: goat anti‐CatE (1:1000; R&D; AF1130); mouse anti‐Aβ (1:1000; Covance; SIG‐39320); rabbit anti‐Aβ (1:1000; ab201060); rabbit anti‐Iba1 (1:1000; Wako; 019–19741); rabbit anti‐GFAP (1:1000; abcam; ab7260); rat anti‐F4/80 (1:2000; abcam; ab6640); and rabbit anti‐p65 (1:1000; abcam; ab16502) at 4°C overnight. Incubation in secondary antibodies was performed for 2h at room temperature before mounting in Vectashield anti‐fading medium (Vector Laboratories). Fluorescent images were taken using a confocal laser scanning microscope (CLSM; Nikon, Japan). The line plot profiles were analyzed using Image J as previously reported (Meng et al., 2020).

### 5.7 Cell culture

The mouse microglial cell line MG6 (RCB2403, Riken BioResource Center,) and mouse neuroblastoma N2a (CCL‐131, ATCC) were maintained in DMEM containing 10% fetal bovine serum (Gibco) supplemented with 2 mg/ml Glucose (Gibco), Penicillin‐Streptomycin (Gibco), 10 μg/ml insulin and 100 M β‐mercaptoethanol (Ni et al., 2015). Primary microglia were prepared from the neonatal cortex in accordance with the previously described methods (Ni et al., 2019).

### 5.8 Cell viability assay

MG6 cells were seeded in 96‐well plates for overnight (5 × 10^3^ cells/well) and then cultured by treatment with different concentrations of Aβ. A cell viability assay was performed using a Cell‐Counting Kit (CCK‐8) (Dojindo, Kumamoto, Japan) according to the previously described methods (Liu et al., 2013). The optical density was read at a wavelength of 450 nm with a microplate reader. Cell viability was calculated using the following formula: optical density of treated group/control group.

### 5.9 Aβ phagocytosis assay

Primary WT and *CatE* ^−/−^ microglia were seeded on 24‐well plates. 1μg/ml HiLyte Fluor 488‐labeled Aβ_1‐42_ (AnaSpec Cat# AS‐60479–01) was added into the culture medium for 6h. The culture medium was collected and centrifuged at 1000g for 10min, and transferred 100 μl to the 96‐well plates. The fluorescent density was determined by microreader (Infinite M200 TECAN).

### 5.10 CatE knock‐down with small interfering RNAs

MG6 cells were seeded on a 6‐well plate at a density of 2 × 10^5^ cells per well in 2ml antibiotic‐free DMEM. After 12h, the cells were transiently transfected with control siRNA‐A (sc‐37007; Santa Cruz Biotechnology) or CatE siRNA (sc‐41474; Santa Cruz Biotechnology), using siRNA Transfection Reagent (sc‐29528; Santa Cruz Biotechnology) according to the manufacturer's protocol. Twelve h after transfection, the cells were subjected Aβ treatment. The cells and conditioned medium at each of the time points were subjected to immunostaining and ELISA analysis.

### 5.11 Real‐time quantitative PCR analysis

Total RNA from tissue and cells were extracted using RNAiso Plus (Takara) and reverse transcribed into cDNA with a QuantiTect Reverse Transcription Kit (Qiagen). Quantitative RT‐PCR was done with Rotor‐Gene SYBR Green Kit with a Corbett Rotor‐Gene RG‐3000A Real‐Time PCR System. mRNA was quantified using the comparative threshold cycle method with beta‐actin as control. Primers used were listed in the Table S2 and Table S3.

### 5.12 Aβ degradation assay

MG6 cells, primary WT or *CatE* ^−/−^ microglia, were seeded into 24‐well plates and allowed to attach overnight. The MG6 cells were transfected with CatE siRNA for 24h, followed by exchanging the medium with serum free DMEM containing 1μg/ml freshly solubilized Aβ_1‐42_ (Anaspec peptide). The concentration of Aβ_1‐42_ in the medium was examined 24h after medium change, which was termed as cell‐based assay. For cell‐free assay, the conditioned medium from MG6 cells or primary microglia were collected and centrifuged at 1000g for 10 min at 48h after seeding. The conditioned medium was then applied into 1.5ml tube mixed with 1μg/ml freshly solubilized Aβ_1‐42_ for 24h. For both assays, the medium at indicated time points were collected and transferred to a tube containing complete protease inhibitor cocktail (Roche).

### 5.13 Luciferase assay

N2a cells were seeded in 24‐well plates at a density of 1x10^5^ cell/ well, the cells were co‐transfected with BACE1 promoter plasmids using HighGene Transfection reagent (ABclonal Technology). The BACE1 promoter plasmids were kindly provided by Prof. Weihui Zhou from Chongqing Medical University. 100 ng/ml sTRAIL were administrated in the cell culture for 24 h. Renilla and firefly luciferase activities were assayed with Dual‐luciferase Reporter Assay System (Promega) according to the manufacturer's instruction.

### 5.14 Enzyme‐linked immunosorbent assay

Soluble proteins from mouse cortical hemispheres were dissolved in TS fraction and insoluble proteins in guanidine‐HCL solution (GuHCL fraction) as reported (Saito et al., 2014). Concentration of Aβ_1‐42_ in the brain lysate and cell culture medium was quantitatively measured by ELISA according to the manufacturer's instructions (296–64401, Wako). Concentration of sTRAIL in the brain lysate and cell culture medium was quantitatively measured by ELISA according to the manufacturer's instructions (RayBiotech).

### 5.15 Stereotactic injection of Aβ

Two‐month‐old WT and *CatE* ^−/−^ mice were anesthetized to full muscle relaxation with ketamine and xylazine (100 mg/kg and 10mg/kg, respectively), and placed in a stereotaxic device. Surgeries were conducted on heated plates, and body temperature was monitored throughout the procedure. The skull was exposed and a hole was drilled into the skull for injection (bregma −2.0 mm, −1.8 mm lateral to midline, 2 mm below the dura). A microsyringe (25G) was used to infuse 1μg Aβ into the brain over 2 min and was left in place for additional 3 min and withdrawn slowly. The incision was closed, and the mice were maintained at 37℃ until fully recovered from anesthesia. 0.2mg/kg buprenorphine was used immediately after surgery to reduce pain.

### 5.16 CatE inhibitor administration

Grassystatin variants (GV), a short grassystatin‐like peptide Ac‐vLN‐Sta‐TAfP‐OH, was synthesized by Shanghai Run Yang Bio Tech. Inc. according to previously reported (Stotz et al., 2020). GV was dissolved in artificial cerebrospinal fluid to a final concentration of 0.15 μM/μl. The cannula (Cat. No. 62064, RWD) was implanted in lateral ventricle (bregma −0.5 mm, ±1.0 mm lateral to midline, 2.3 mm below the dura), and mice were sent back to home cages (one mouse per cage) and recovered over one week. Steel internal cannula (Cat. No. 62264, RWD) projecting 0.5 mm beyond the tip of the guide cannula was connected to a syringe pump (Cat. No. UMP3‐1, WPI). The inhibitor was infused in a volume of 1 μL at a rate of 100 nL per minute and the internal cannula was withdrawn 10 min after the end of infusion. The inhibitor was administered once every three days for a total of seven doses, and the Y‐maze behavior test was performed two hours after the last dose.

### 5.17 Y‐maze test

The apparatus consisted of 3 identical black Plexiglas arms (40 × 10 × 20 cm; Shinfactory, Fukuoka, Japan). Each mouse was placed at the end of one fixed arm and allowed to move freely through the maze during a five‐min session. The sequence of arm entries was recorded visually, and three consecutive choices were defined as an alternation.

% alteration = (Number of alterations/Number of entries) × 100. In addition, the total number of arms entered during the session was also determined.

### 5.18 Quantification morphological analyses of microglia

Confocal Z stack images were captured from the brain of AD mice with or without GV administration. Soma of microglia were quantified as reported previously (Ni et al., 2019). The morphological analyses of microglia were performed using Z‐projections of confocal images. Microglial processes were traced and reconstructed as a single microglia image using the Simple Neurite Tracer program, and the total process length was semi‐automatically traced using three‐dimensional image data.

### 5.19 Measurement of CatE enzymatic activity

Enzymatic activity of CatE was measured using a fluorescence‐quenching substrate (MOCAc‐Gly‐Ser‐Pro‐Ala‐Phe‐Leu‐Ala‐Lys(Dnp)‐D‐Arg‐NH2, Peptide Institute, Inc). Reaction mixtures contained 80 μl of 50mM sodium acetate buffer, pH 4.0, 10 μl of 200 μM substrate solution, and 10 μl of sample solution containing 2 μl of sera and 0.1% Triton X‐100. After 10 min incubation at 40℃, the reaction was terminated by adding 200μl of 150mM Tris‐HCl buffer, pH 8.8. The increase in fluorescence intensity produced by substrate cleavage was measured at an emission wavelength of 405 nm with excitation at 320nm using a microplate reader Infinite M200pro Spectrophotometer (Tecan, Switzerland). The data represent the relative value of CatE enzymatic activity that normalized to ACSF‐treated mice.

### 5.20 Statistical analysis

All data are representative of at least three different experiments. Data are means ±SEM of three independent experiments. The statistical analyses were performed by student's t‐test, one‐way ANOVA with a post hoc Tukey's test using the GraphPad Prism software package (GraphPad Software). A value of *p *< 0.05 was considered to indicate statistical significance.

## CONFLICT OF INTERESTS

None declared.

## AUTHORS’ CONTRIBUTION

Z.X., J.M., and W.K. performed experiments, analyzed data, and wrote the paper. Z.W., F.L., and N. performed some experiments and analyzed data. Y.H., Q.Y., Z.B., and H.N. provided samples or reagents and analyzed data. H.Q. designed part of the experiments. J.N. supervised this research, analyzed data, and wrote the paper.

## Supporting information

Fig S1‐S10

Supplementary Material

## Data Availability

All data needed to evaluate the conclusions in the paper are present in the paper and/or the Supplementary Materials.
