# Supplementary figures and images for: Microglial cathepsin E plays a role in neuroinflammation and amyloid β production in Alzheimer’s disease
Source: Aging Cell. 2022 Feb 19;21(3):e13565. doi: 10.1111/acel.13565 (PMC8920437; doi:10.1111/acel.13565)

**
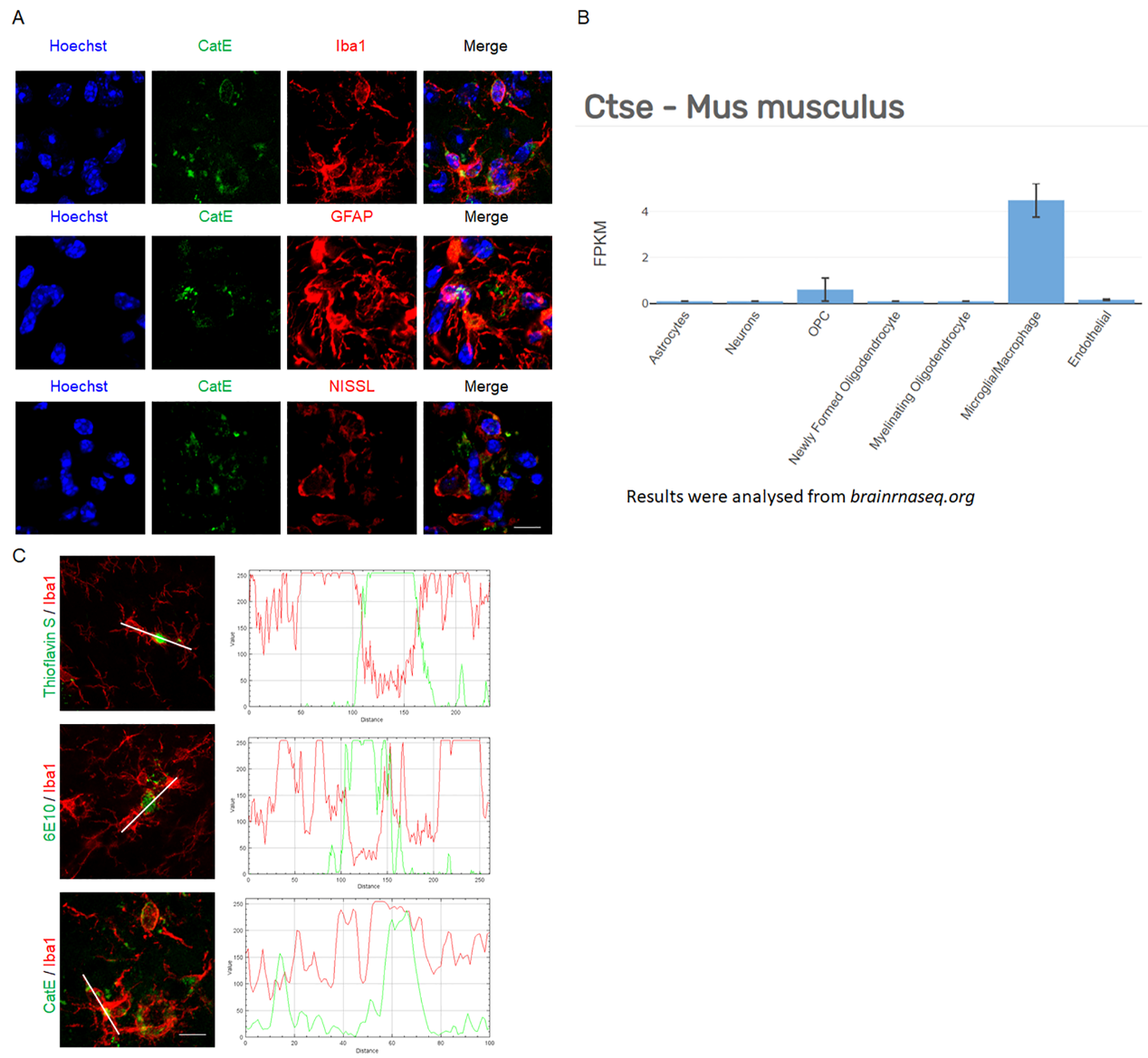
Figure.S1**

**Figure.S2**

**
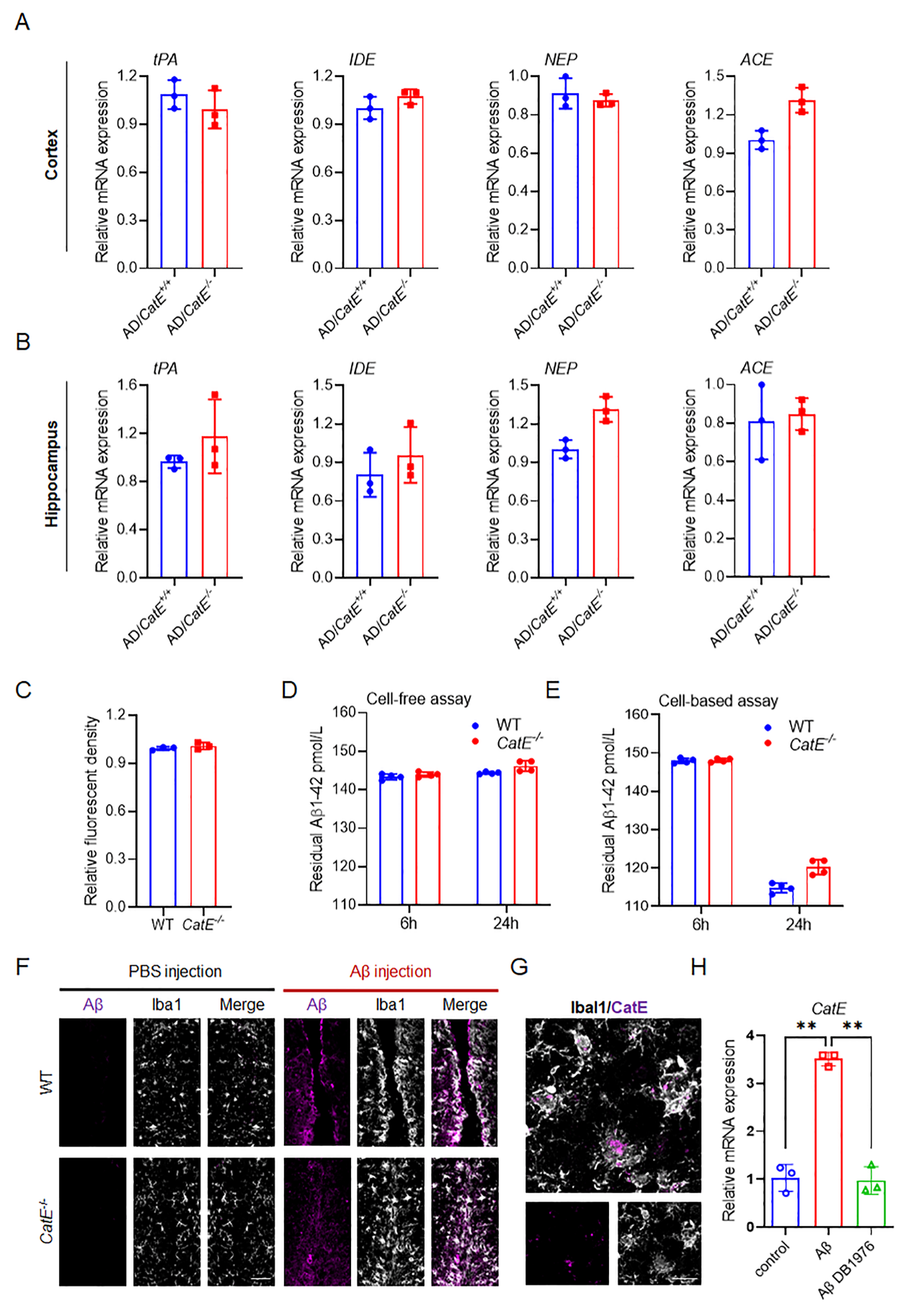
**

**Figure.S3**

**
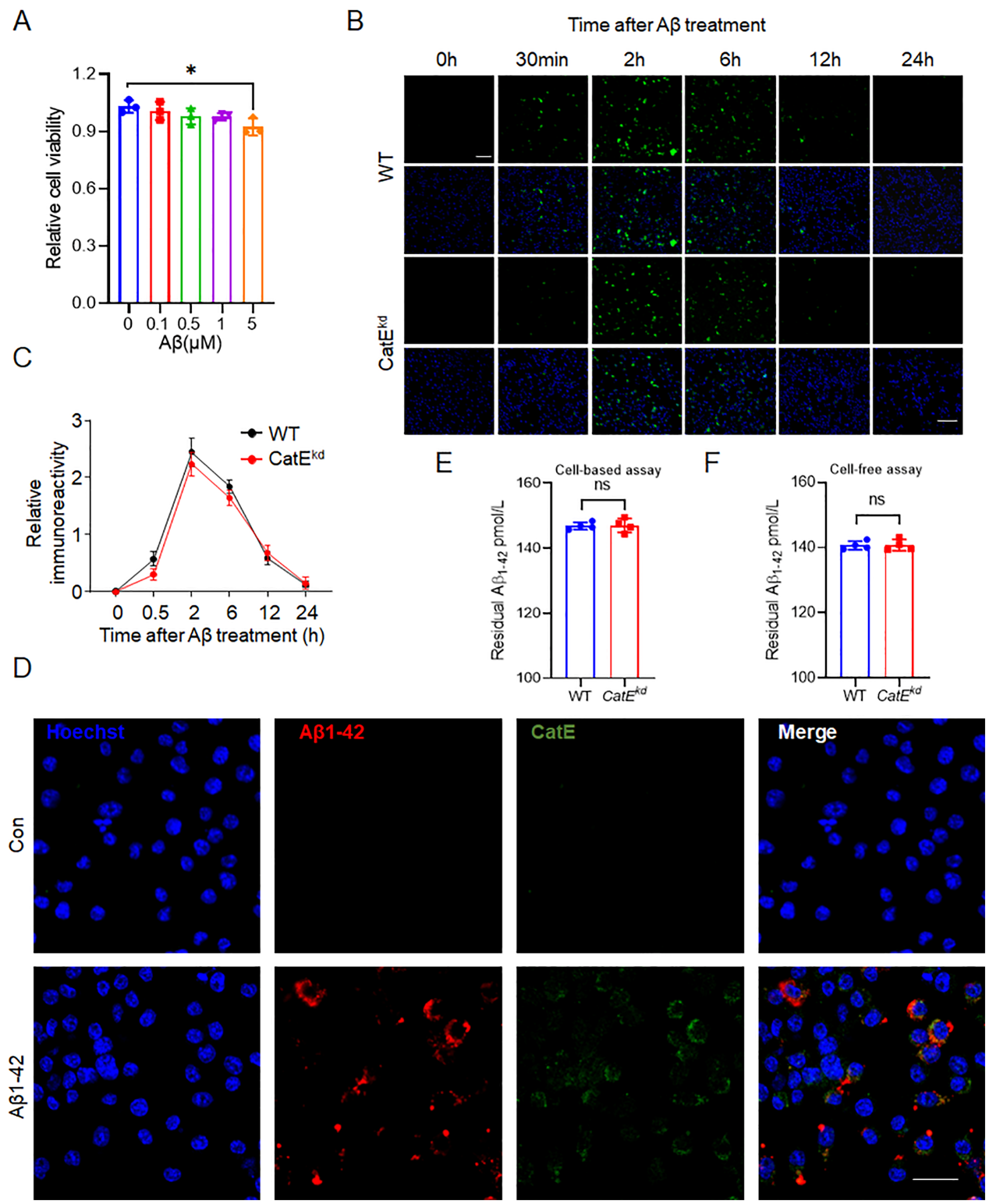
**

**Figure.S4**

**
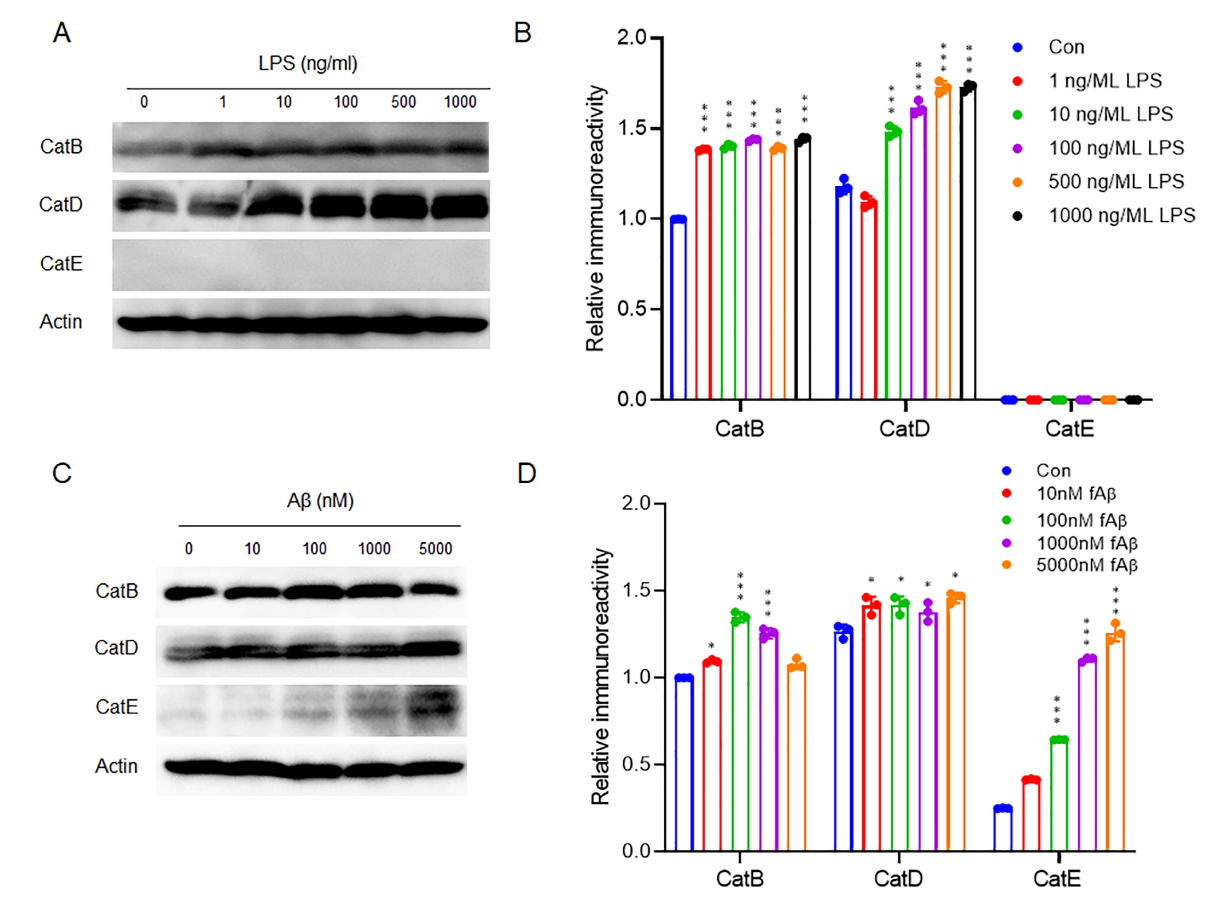
**

**Figure.S5**

**
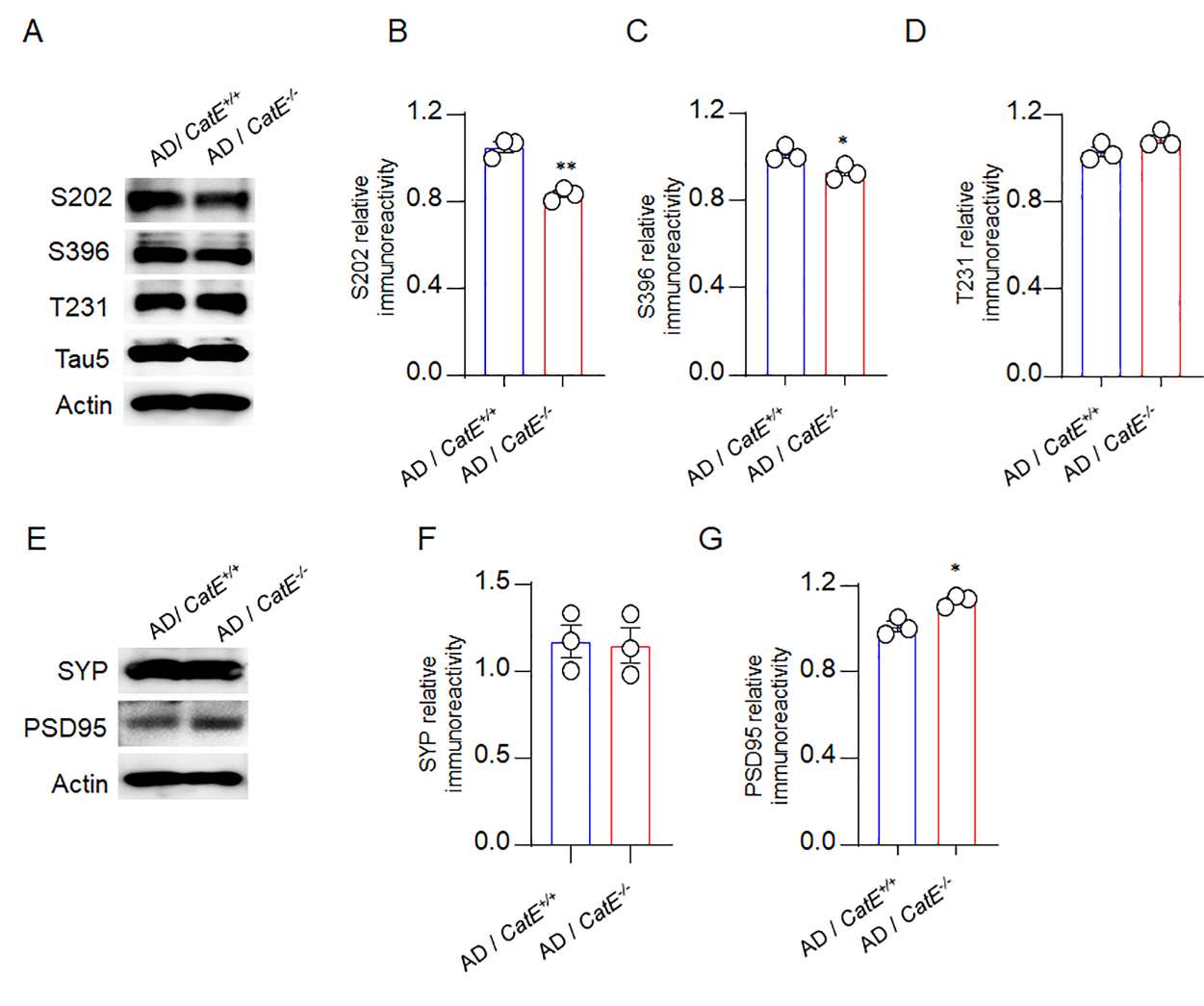
**


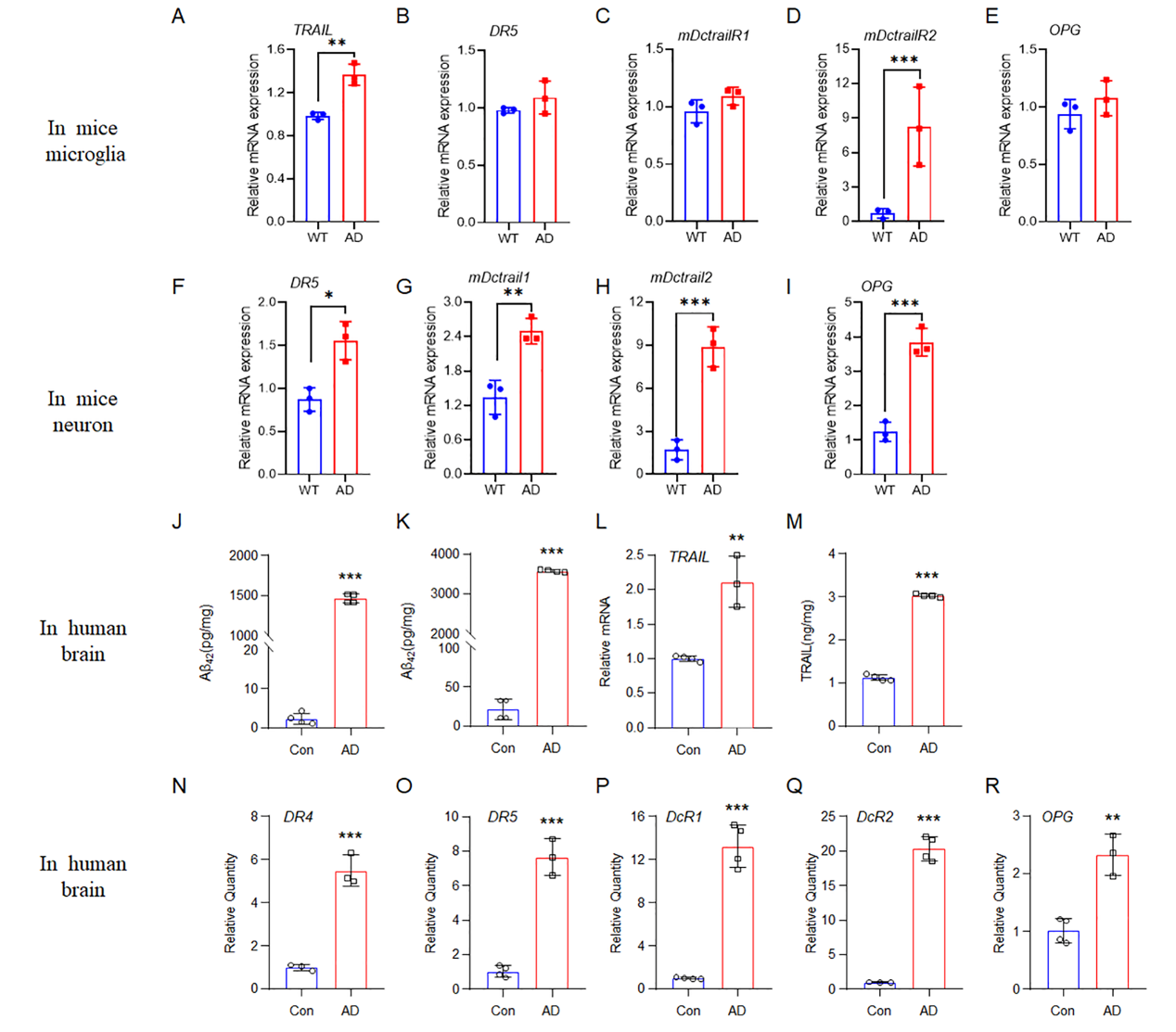
**Figure.S6**


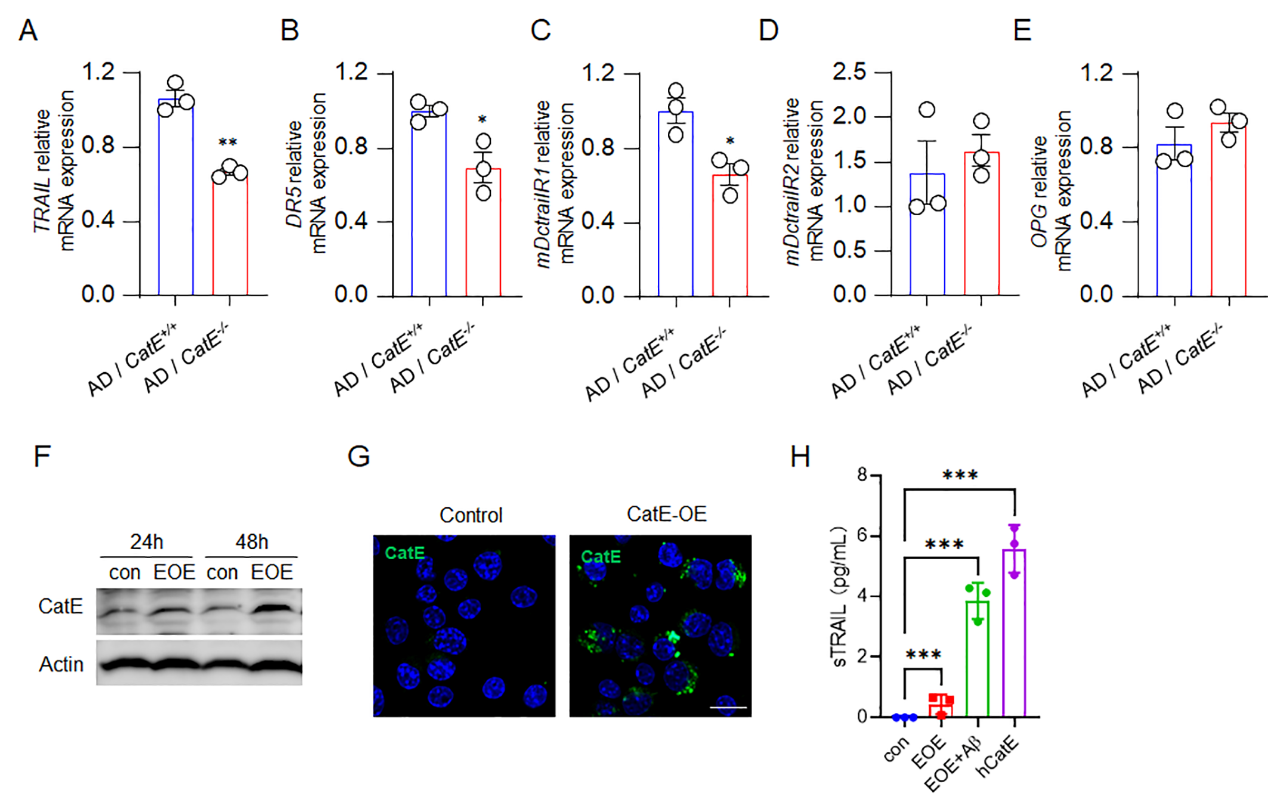
**Figure.S7**

**Figure.S8**

**
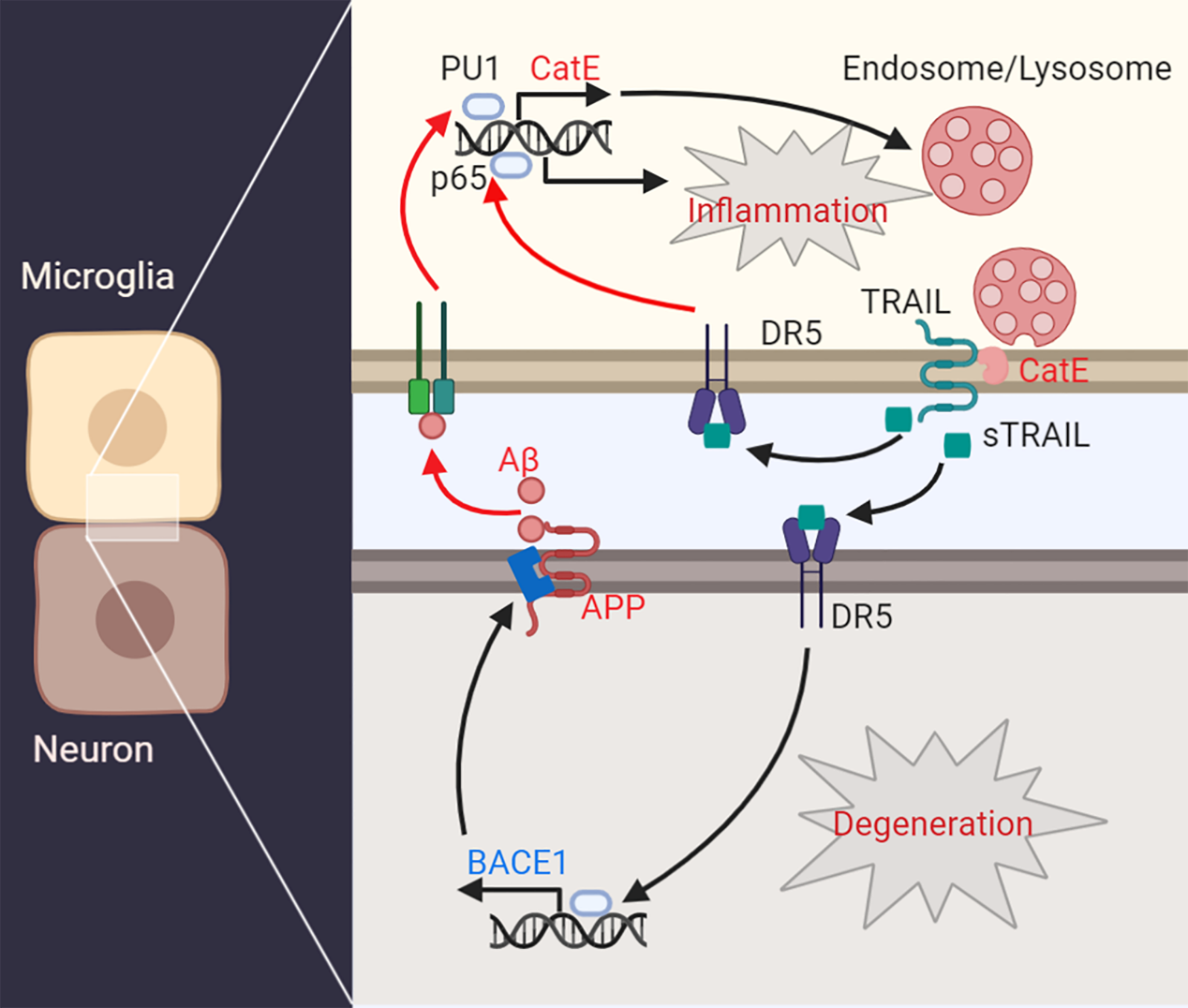
**

**Figure.S9**

**
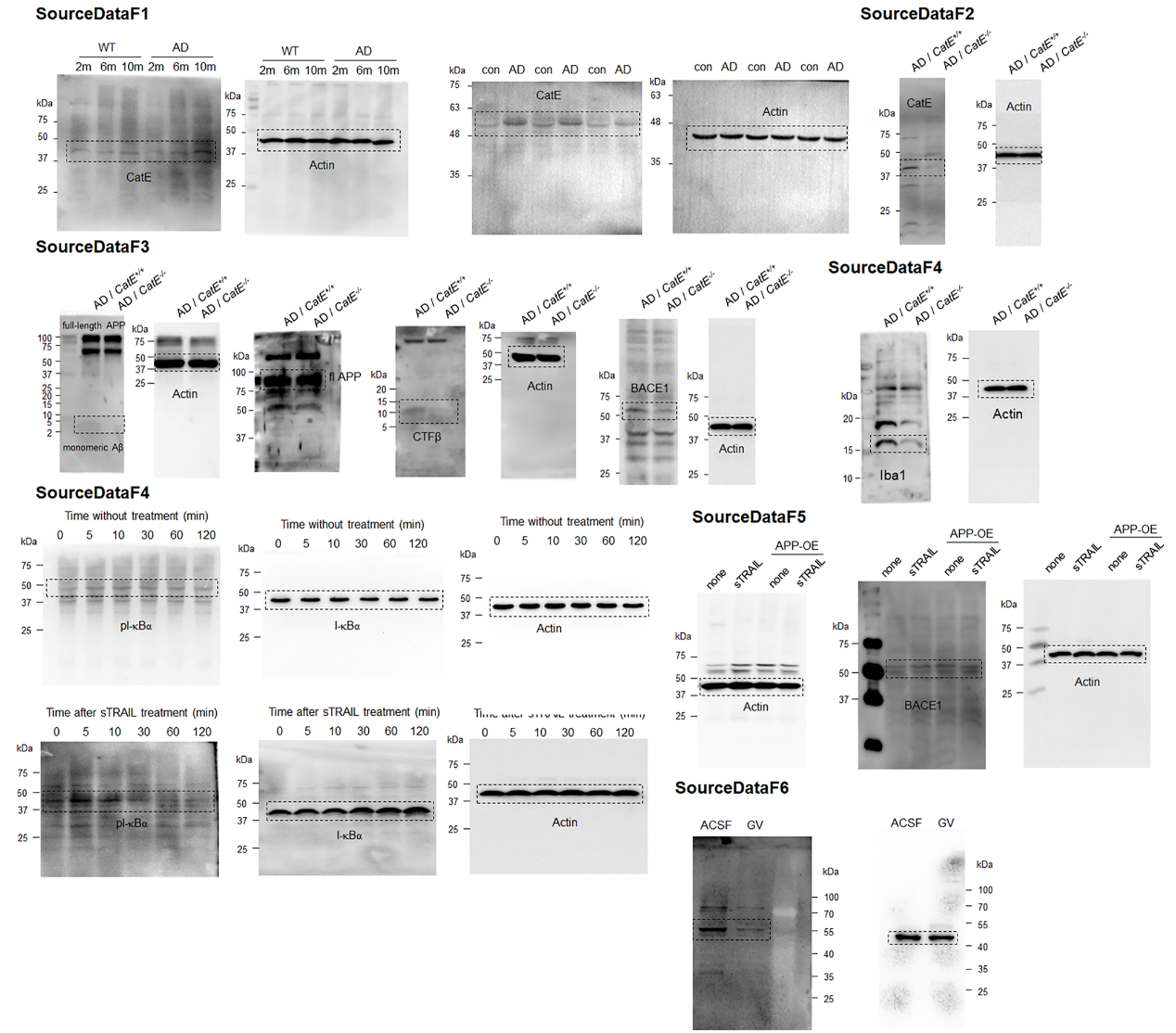
**

**Figure.S10**

**
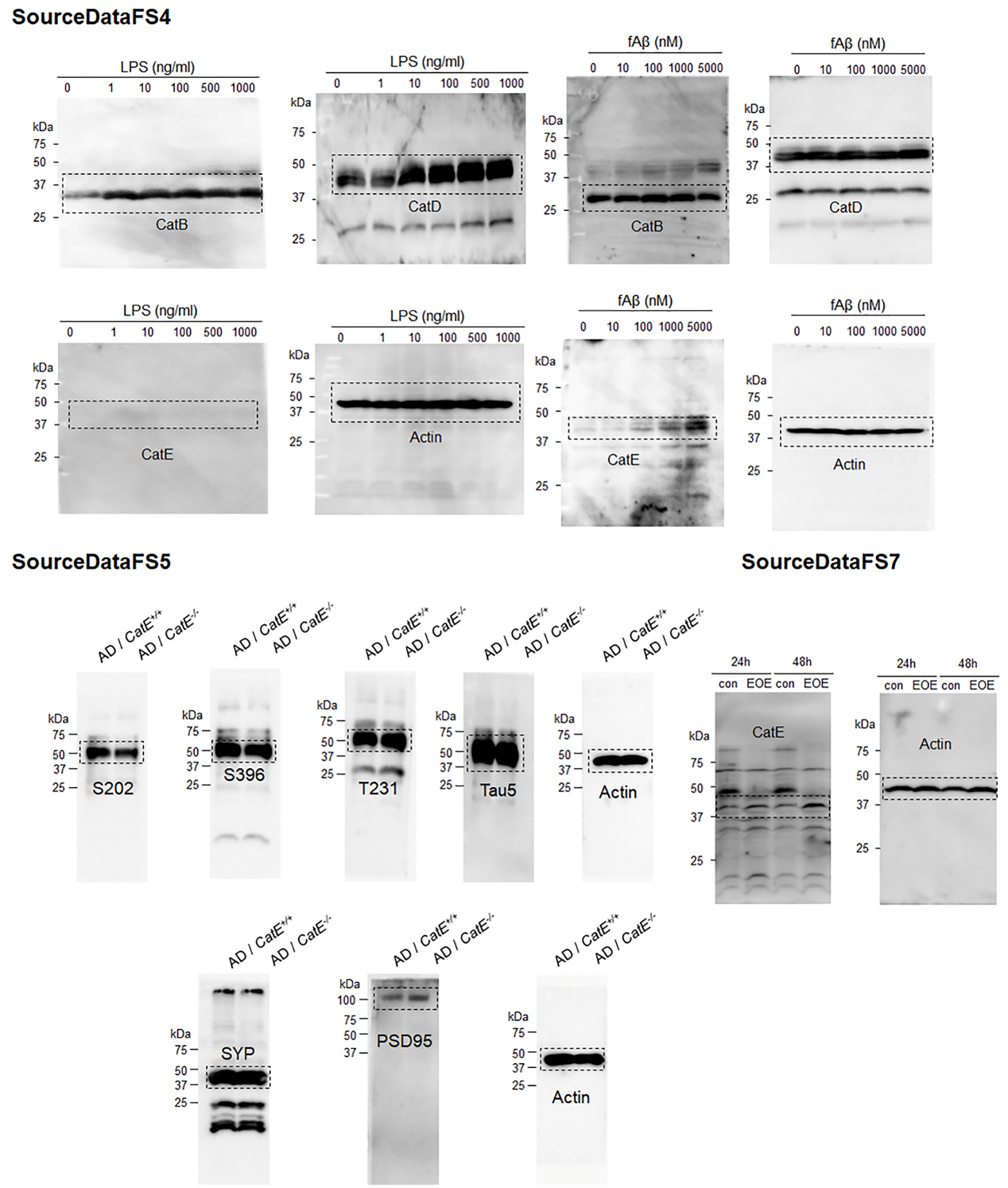
**

Supplement: Supplementary file 1 — Fig S1‐S10 [file ACEL-21-e13565-s001.docx]
