# Supplementary material for: Microglial cathepsin E plays a role in neuroinflammation and amyloid β production in Alzheimer’s disease
Source: Aging Cell. 2022 Feb 19;21(3):e13565. doi: 10.1111/acel.13565 (PMC8920437; doi:10.1111/acel.13565)
Supplement: Supplementary file 2 — Supplementary Material [file ACEL-21-e13565-s002.docx]

**SUPPLEMENTAL INFORMATION**

**Microglial Cathepsin E Plays a Role in Neuroinflammation and Amyloid β Production in Alzheimer’s Disease**

Zhen Xie^1*^, Jie Meng^2, 3*^, Wei Kong^1*^, Zhou Wu^3^, Fei Lan^1^, Narengaowa^1^,

Yoshinori Hayashi^4^, Qinghu Yang^5^, Zhantao Bai^5^, Hiroshi Nakanishi^6^, Hong Qing^1†^,

Junjun Ni^1,^ ^3†^

**Supplemental experimental procedures**

**Cell culture**

The mouse microglial cell line MG6 (RCB2403, Riken BioResource Center,), and mouse neuroblastoma N2a (CCL-131, ATCC) were maintained in DMEM containing 10% fetal bovine serum (Gibco) supplemented with 2 mg/ml Glucose (Gibco), Penicillin-Streptomycin (Gibco), 10 μg/ml insulin and 100 M β-mercaptoethanol (Ni et al., 2015). Primary microglia were prepared from the neonatal cortex in accordance with the previously described methods (Ni et al., 2019).

**Cell isolation**

Six-months-old WT and AD mouse brains were collected and enzymatically digested using the Neural Tissue Dissociation Kit (Miltenyi Biotec) at 37°C. Further processing was performed at 4°C. Tissue debris were removed by passing the cell suspension through a 30μm cell strainer and magnetically labelled with CD11b microbeads, the cells were extensively washed and separated in a magnetic field using MS columns (Miltenyi Biotec), and the CD11b-positive fraction was collected. CD11b-negative fraction was collected for further neuron purification using Neuron Isolation Kit, mouse (Miltenyi Biotec).

**Cell viability assay**

MG6 cells were seeded in 96-well plates for overnight (5×10^3^ cells/well) and then cultured by treatment with different concentrations of Aβ. A cell viability assay was performed using a Cell-Counting Kit (CCK-8) (Dojindo, Kumamoto, Japan) according to the previously described methods (Liu et al., 2013). The optical density was read at wavelength of 450 nm with a microplate reader. Cell viability was calculated using the following formula: optical density of treated group/control group.

**CatE knockdown with small interfering RNAs.**

MG6 cells were seeded on a 6-well plate at a density of 2×10^5^ cells per well in 2ml antibiotic-free DMEM. After 12h, the cells were transiently transfected with control siRNA-A (sc-37007; Santa Cruz Biotechnology) or CatE siRNA (sc-41474; Santa Cruz Biotechnology), using siRNA Transfection Reagent (sc-29528; Santa Cruz Biotechnology) according to the manufacturer’s protocol. Twelve hours after transfection, the cells were subjected Aβ treatment. The cells and conditioned medium at each of the time points were subjected to immunostaining and ELISA analysis.

**Aβ phagocytosis assay**

Primary WT and *CatE^-/-^* microglia were seeded on 24 well plates. 1μg/mL HiLyte Fluor 488-labeled Aβ_1-42_ (AnaSpec Cat# AS-60479-01) was added into the culture medium for 6h. The culture medium was collected and centrifuged at 1000g for 10min, and transferred 100 μl to the 96-well plates. The fluorescent density was determined by microreader (Infinite M200 TECAN).

**Enzyme-linked immunosorbent assay**

Soluble proteins from mouse cortical hemispheres were dissolved in TS fraction and insoluble proteins in guanidine-HCL solution (GuHCL fraction) as reported (Saito et al., 2014). Concentration of Aβ_1-42_ in the brain lysate and cell culture medium were quantitatively measured by ELISA according to the manufacturer’s instructions (296-64401, Wako). Concentration of sTRAIL in the brain lysate and cell culture medium were quantitatively measured by ELISA according to the manufacturer’s instructions (RayBiotech).

**Aβ degradation assay**

MG6 cells, primary WT or *CatE^-/-^* microglia were seeded into 24-well plates and allowed to attach overnight. The MG6 cells were transfected with CatE siRNA for 24h, followed by exchanging the medium with serum free DMEM containing 1μg/mL freshly solubilized Aβ_1-42_ (Anaspec peptide). The concentration of Aβ_1-42_ in the medium were examined 24h after medium change, which was termed as cell-based assay. For cell-free assay, the conditioned medium from MG6 cells or primary microglia were collected and centrifuged at 1000g for 10 min at 48h after seeding. The conditioned medium was then applied into 1.5ml tube mixed with 1μg/mL freshly solubilized Aβ_1-42_ for 24h. For both assays, the medium at indicated time points were collected and transferred to a tube containing complete protease inhibitor cocktail (Roche).

**Luciferase assay**

N2a cells were seeded in 24-well plates at a density of 1x10^5^ cell/ well, the cells were co-transfected with BACE1 promoter plasmids using HighGene Transfection reagent (ABclonal Technology). The BACE1 promoter plasmids were kindly provided by Prof. Weihui Zhou from Chongqing Medical University. 100 ng/mL sTRAIL were administrated in the cell culture for 24 h. Renilla and firefly luciferase activities were assayed with Dual-luciferase Reporter Assay System (Promega) according to the manufacturer’s instruction.

**Tissue collection and sample preparation**

Mice were anesthetized with somnopentyl (50 mg/kg, Kyoritsu Seiyaku) and perfused with ice-cold PBS. The brain was bisected for biochemical analysis and histological analysis. The cortices and hippocampi were carefully dissected out under a dissection microscope. Tissues for biochemical analysis were stored in -80℃ before further processing and tissues for histological analysis were fixed in 4 % PFA overnight followed by submergence in 30% sucrose before freezing.

**Immunoblotting analyses**

Human and mouse brain and cultured cells were homogenized in RIPA buffer and the samples in equal amount of protein were loaded on 7.5, 12, and 15% SDS-polyacrylamide gels and then transferred to 0.4μm PVDF membranes. Specially, the level of soluble Aβ were analyzed using tricine-SDS-PAGE and transferred to 0.2μm PVDF membranes according to the published methods (Schagger, 2006). The blots were probed with the following antibodies: goat anti-CatE (1:1000; R&D; AF1130); goat anti-CatE (1:1000; R&D, AF1294); mouse anti-CatD (1:1000; Santa Cruz Biotechnology, sc-377299); mouse anti-CatB (1:1000; Santa Cruz Biotechnology, sc-365558); mouse anti-Aβ (1:1000; Covance; SIG-39320); rabbit anti-APP C-Terminal (1:1000; Millipore; 171610); mouse anti BACE1 (1:1000; Millipore; MAB5308); goat anti IL-1β (1:1000; R&D; AF-401-NA); rabbit anti-Iba1 (1:1000; Wako019-19741); rabbit anti-post synaptic protein 95 (PSD95) (1:2000; abcam; ab18258); rabbit anti-synaptophysin (SYP) (1:2000; abcam; ab14692); rabbit anti-S202 (1:5000; abcam; ab108387); rabbit anti-S396 (1:5000; abcam; ab109390); anti-T231(1:5000; abcam; ab151559); mouse anti-Tau5 (1:5000; abcam; ab80579); rabbit anti-IκBα (1:1000; Cell Signaling Technology; Cat# 8993); mouse anti-pIκBα (1:1000; Cell Signaling Technology; Cat# 9246) and mouse anti-actin (1:5000; abcam; ab49900). Primary antibodies were incubated overnight at 4°C and followed by incubation with second antibodies for 1h at room temperature before washing by TBST for 3 times. The HRP-labeled antibodies were detected by an ECL kit with image analyzer (LAS-41000; Fuji Photo Film).

**Immunofluorescent staining**

Brain sections and fixed cells were blocked in the blocking buffer (3% BSA, and 0.3% Triton X-100 in PBS) for 1h at room temperature and then incubated with primary antibodies: goat anti-CatE (1:1000; R&D; AF1130); mouse anti-Aβ (1:1000; Covance; SIG-39320); rabbit anti-Aβ (1:1000; ab201060); rabbit anti-Iba1 (1:1000; Wako; 019-19741); rabbit anti-GFAP (1:1000; abcam; ab7260); rat anti-F4/80 (1:2000; abcam; ab6640) and rabbit anti-p65 (1:1000; abcam; ab16502) at 4°C overnight. Incubation in secondary antibodies was performed for 2h at room temperature before mounting in Vectashield anti-fading medium (Vector Laboratories). Fluorescent images were taken using a confocal laser scanning microscope (CLSM; Nikon, Japan). The line plot profiles were analyzed using Image J as previously reported (Meng et al., 2020).

**Stereotactic injection of Aβ**

Two-month old WT and *CatE^-/-^* mice were anesthetized to full muscle relaxation with ketamine and xylazine (100 mg/kg and 10mg/kg, respectively) and placed in a stereotaxic device. Surgeries were conducted on heated plates and body temperature was monitored throughout the procedure. The skull was exposed and a hole was drilled into the skull for injection (bregma -2.0 mm, -1.8 mm lateral to midline, 2 mm below the dura). A microsyringe (25G) was used to infuse 1μg Aβ into the brain over 2 min and was left in place for additional 3 min and withdrawn slowly. The incision was closed and the mice were maintained at 37℃ until fully recovered from anesthesia. 0.2mg/kg buprenorphine was used immediately after surgery to reduce pain.

**Quantification morphological analyses of microglia**

Confocal Z stack images were captured from the brain of AD mice with or without GV administration. Soma of microglia were quantified as reported previously (Ni et al., 2019). The morphological analyses of microglia were performed using Z-projections of confocal images. Microglial processes were traced and reconstructed as a single microglia image using the Simple Neurite Tracer program, and the total process length was semi-automatically traced using three-dimensional image data.

**Statistical analysis**

All data are representative of at least three different experiments. Data are means ± SEM of three independent experiments. The statistical analyses were performed by student’s t-test, one-way ANOVA with a post hoc Tukey’s test using the GraphPad Prism software package (GraphPad Software). A value of *p*<0.05 was considered to indicate statistical significance.

**Supplemental figure legends**

**Figure S1.** CatE was majorly expressed in the microglia of AD mice. (**A**), Immunofluorescent images of CatE (green) with Iba1 (red), GFAP (red) and NISSL (red) in the hippocampus from 6-month old AD mice. Scale bar, 10μm. (**B**), RNAseq indicated microglia/macrophage were the major CatE-expressed cell types. Data were analysed from *brainrnaseq.org*. (**C**), Immunofluorescent images of Iba1(red) with Thioflavin S (green), Iba1 (red) and Aβ (green), and Iba1(red) with CatE (green) in the hippocampus from 6-month old AD mice. Scale bar, 10μm. The profile plots fluorescence intensity (values from 0 to 250) of Iba1 (red) and Thioflavin S, 6E10 or CatE (green) at the position along the white lines in the left corresponding images.

**Figure S.2** CatE is not involved in degradation of Aβ. (**A, B**), Relative mRNA expression of tPA, IDE, NEP and ACE in the cortex (A) and hippocampus (B) from 6-month old AD mice and AD/*CatE^-/-^* mice. Values are mean ± SEM (n=3 mice/genotype). (**C**), Relative fluorescent density of phagocytic Aβ-FITC in WT and *CatE^-/-^* primary microglia. Values are mean ± SEM (n=3 cell culture preparations). (**D, E**), Aβ degradation assay using the cell-free assay (D) and cell-based assay (E) using WT and *CatE^-/-^* primary microglia. Values are mean ± SEM (n=4 cell culture preparations). (**F**), Immunofluorescent images of Aβ (magenta) with Iba1 (white) in the cortex from 2-month old WT and *CatE^-/-^* mice after Stereotactic injection of Aβ or PBS for 5 days. Scale bar, 50μm. (**G**), Immunofluorescent images of CatE (magenta) with Iba1 (white) in the cortex from 2-month old WT mice after Stereotactic injection of Aβ for 5 days. Scale bar, 20μm. (**H**), Relative mRNA expression of CatE in MG6 cells after Aβ treatment in the presence or absence of DB1976. Values are mean ± SEM (n=3 independent experiments). **P<0.01, One-way ANOVA.

**Figure S3.** Phagocytic and degradative activity of Aβ do not change in MG6 cells after CatE knockdown. (**A**), Relative cell viability of MG6 cells after treatment with 0, 0.1, 0.5, 1 and 5μM Aβ for 24h. Values are mean ± SEM (n=3 cell culture preparations). *P<0.05, One-way ANOVA. (**B**), Immunofluorescent images of Aβ (green) with Hoechst (blue) in the MG6 with or without CatE knockdown after application with 1μM Aβ for 0, 0.5, 2, 6, 12 and 24h. Scale bar, 100μm. (**C**), Quantification of the relative immunofluorescent density of Aβ shown in (B). Values are mean ± SEM (n=3 cell culture preparations). (**D**), Immunofluorescent images of Aβ (red) with CatE (green) and Hoechst (blue) in the MG6 after application with 1μM Aβ for 6h. Scale bar, 20μm. (**E, F**), Aβ degradation assay using the cell-based assay (E) and cell-free assay (F) using MG6 cells with or without CatE knockdown. Values are mean ± SEM (n=4 cell culture preparations). ns, no significant difference.

**Figure S4.** Differential expression of CatE compared with CatB and CatD. (**A**), Immunoblot analysis of CatB, CatD and CatE in MG6 cells after treatment with 0, 1, 10, 100, 500, 1000 ng/mL LPS for 48 h. (**B**), Quantification of CatB, CatD and CatE in the immunoblots shown in (A). Values are mean ± SEM (n=3 cell culture preparations). ***P<0.001, One-way ANOVA. (**C**), Immunoblot analysis of CatB, CatD and CatE in MG6 cells after treatment with 0, 1, 10, 100, 1000 and 5000 nM Aβ for 48 h. (**D**), Quantification of CatB, CatD and CatE in the immunoblots shown in (C). Values are mean ± SEM (n=3 cell culture preparations). *P<0.05, **P<0.01, ***P<0.001, One-way ANOVA.

**Figure S5.** CatE deficiency ameliorates the Tau phosphorylation and synaptic protein reduction in AD mice. (**A**), Immunoblot analysis of S202, S396, T231 and Tau5 in the hippocampus of from 6-month old AD and AD /*CatE^-/-^* mice. (**B-D**), Quantification of S202 (B), S396 (C) and T231 (D) in the immunoblots shown in (A). Values are mean ± SEM (n=3 mice/genotype). *P<0.05, **P<0.01, Student’s t-test. (**E**), Immunoblot analysis of synaptophsin (SYP) and post synaptic protein 95 (PSD95) in the hippocampus of from 6-month old AD and AD/*CatE^-/-^* mice. (**F, G**), Quantification of SYP (F) and PSD95 (G) in the immunoblots shown in (E). Values are mean ± SEM (n=3 mice/genotype). *P<0.05, Student’s t-test.

**Figure S6.** Expression of TRAIL and its receptors in microglia and neurons from isolated from 6-month old AD mice. (**A-E**), Relative mRNA expression of TRAIL(A), DR5(B), mDctrailR1(C), mDctrailR2(D) and OPG(E) in cortical microglia from 6-month old WT and AD mice. Values are mean ± SEM (n=3 mice/genotype). ***P<0.001, Student’s t-test. (**F-I**), Relative mRNA expression of DR5(F), mDctrailR1(G), mDctrailR2(H) and OPG(I) in cortical neuron from 6-month old WT and AD mice. Values are mean ± SEM (n=3 mice/genotype). ***P<0.001, Student’s t-test. (**J, K**), The amount of soluble (J) and insoluble Aβ (K) in the brain lysates from AD patients and age-matched control. (**L, M**), Relative mRNA (L) and protein amount (M) of TRAIL in the cortex from AD patients and age-matched control. (**N-R**), Relative mRNA expression of DR4, DR5, DcR1, DcR2 and OPG in the in the brain from AD patients and age-matched control. Values are mean ± SEM (n=3-4 samples). **P<0.01, ***P<0.001, Student’s t-test.

**Figure S7.** Expression of TRAIL and its receptors in in the hippocampus of from 6-month old AD and AD/*CatE^-/-^* mice. (**A-E**), Relative mRNA expression of TRAIL(A), DR5(B), mDctrailR1(C), mDctrailR2(D) and OPG(E) in cortex from 6-month old AD and AD/*CatE^-/-^* mice. Values are mean ± SEM (n=3 mice/genotype). *P<0.05, Student’s t-test. (**F**), Immunoblot analysis of CatE in MG6 cells after overexpression of CatE for 24 and 48h. (**G**), Immunofluorescent images of CatE (green) with Hoechst (blue) in the MG6 microglia cells after overexpression of CatE for 48h. Scale bar, 10μm. (**H**), Amount of sTRAIL in the cell culture medium from MG6 cells after overexpression of CatE for 48h or combination with 1μM Aβ for 24h or treatment with human recombinant CatE for 48h. Values are mean ± SEM (n=3 cell culture preparations). ***P<0.001, One-way ANOVA.

**Figure S8.** Schematic illustration of the CatE-TRAIL axis on neuroinflammation and Aβ production. The black arrows indicate the major finding in the present study.

**Figure S9.** The full-length blot for figures in the main text.

**Figure S10.** The full-length blot for supplementary figures in the supplementary materials.

**Table S1.** Primer sequences of genes used for genotyping.

**Table S2.** Primer sequences of mouse genes used for real-time PCR.

**Table S3.** Primer sequences of human genes used for real-time PCR.

**Table S1: Primer sequences of genes used for genotyping.**

| Gene | Primers sequences | |
| --- | --- | --- |
| *APP^NL-G-F^* | Forward1 | 5′-ATCTCGGAAGTGAAGATG-3′ |
|  | Reverse1 | 5′-ATCTCGGAAGTGAATCTA-3′ |
|  | Forward2 | 5′-TGTAGA TGAGAACTTAAC-3′ |
|  | Reverse2 | 5′-CGTATAATGTATGCTATACGAAG-3′ |
|  | S1 | 5’-AGGGTGGGGTTGATGGTAAG-3’ |
| *CatE* | W1 | 5’-TGAAAATGAGGGTGTTGAGGT-3’ |
|  | N1 | 5’-TGGCTGCTATTGGGCGAAGTG-3’ |

**Table S2: Primer sequences of mouse genes used for real-time PCR**

| Gene | Primers sequences | |
| --- | --- | --- |
| *BACE1* | Forward | 5’-GATGGTG GACAACCTGAG-3’ |
|  | Reverse | 5’-CTGGTAGTAGCGATGCAG-3’ |
| *IL-1β* | Forward | 5’-CAACCAACAAGTGATATTCTCCATG-3’ |
|  | Reverse | 5’-GATCCACACTCTCCAGCTGCA-3’ |
| *TNF-α* | Forward | 5’-CTGTAGCCCACGTCGTAGC-3’ |
|  | Reverse | 5’-TTGAGATCCATGCCGTTG-3’ |
| *NOS2* | Forward | 5’-GCCACCAACAATGGCAAC-3’ |
|  | Reverse | 5’-CGTACCGGATGAGCTGTGAATT-3’ |
| *IL-6* | Forward | 5’-TCAATTCCAGAAACCGCT ATGA-3’ |
|  | Reverse | 5’-CACCAGCATCAGTCCCAAGA-3’ |
| *CatE* | Forward | 5’-CAG TCCGACACATACACG-3’ |
|  | Reverse | 5’-TGGCCTGGCTCCTTGAC-3’ |
| *IDE* | Forward | 5’-CCGAAAGCTTGTCTCAACTT-3’ |
|  | Reverse | 5’-ATACATCCCATAGATGGTATTTTGG-3’ |
| *NEP* | Forward | 5’-TCCTGACTATCATAGCGGTGAC-3’ |
|  | Reverse | 5’-GACGTTGCGTTTCAACCAGC-3’ |
| *ACE* | Forward | 5’-TGGGCAAACTCTATGCTG-3’ |
|  | Reverse | 5’-TTCATTGGCTCCGTTTCTTA-3’ |
| *tPA* | Forward | 5’-TGCCTTCCTCTTCCTCTTCTACAG-3’ |
|  | Reverse | 5’-AGATACCCCTTCCTTCTCGTGG-3’ |
| *Iba1* | Forward | 5’-CAGACTGCCAGCCTAAGACA-3’ |
|  | Reverse | 5’-AGGAATTGCTTGTTGATCCC-3’ |
| *CD86* | Forward | 5’-TCAATGGGACTGCATATCTGCC-3’ |
|  | Reverse | 5’-GCCAAAATACTACCAGCTCACT-3’ |
| *Trail* | Forward | 5’-GACCAGCTCTCCATTCCTA-3’ |
|  | Reverse | 5’-GAAGACCTCAGAAAGTGGC-3’ |
| *DR5* | Forward | 5’-AAGTGTGTCTCCAAAACGG-3’ |
|  | Reverse | 5’-AATGCACAGAGTT CGCACT-3’ |
| *mDctrailR1* | Forward | 5’-AGGAATGCAACTCCACAGCTAAC-3’ |
|  | Reverse | 5’-TTGCCTCCATGGTTTCTCTTCAC-3’ |
| *mDctrailR2* | Forward | 5’-CCCATACTCAAGGACAATGTGAG-3’ |
|  | Reverse | 5’-GCACGATTCTGGAAATTTTGGG-3’ |
| *OPG* | Forward | 5’-CAGCTCACAAGAGCAAACCTTCCA-3’ |
|  | Reverse | 5’-ACGCTGCTTTCACAGAGGTCAA-3’ |
| *Actin* | Forward | 5’-AGAGGGAAATCGTGCGTGAC-3’ |
|  | Reverse | 5’-CAATAGTGATGACCTGGCCGT-3’ |

**Table S3: Primer sequences of human genes used for real-time PCR**

| Gene | Primers sequences | |
| --- | --- | --- |
| *APP* | Forward | 5’-GATGCGGAGGAGGATGAC-3’ |
|  | Reverse | 5’-TCTGTGGCTTCTTCGTAGG-3’ |
| *TRAIL* | Forward | 5’-TTGTTGATGAAAAGTGCTAGAAATA-3’ |
|  | Reverse | 5’-ATGGTCCATGTCTATCAAGTGC-3’ |
| *DR4* | Forward | 5’-CTGAGCAACGCAGACTCGCTGTCCAC-3’ |
|  | Reverse | 5’-TCAAAGGACACGGCAGAGCCTGTGCCA-3’ |
| *DR5* | Forward | 5’-GGGAGCCGCTCATGAGGAAGTTGG-3’ |
|  | Reverse | 5’-GGCAAGTCTCTCTCCCAGCGTCTC-3’ |
| *DcR1* | Forward | 5’-CCCTAAAGTTCGTCGTCGTCAT-3’ |
|  | Reverse | 5’-GGGCAGTGGTGGCAGAGTA-3’ |
| *DcR2* | Forward | 5’-GTTGGCTTTTCATGTCGGAAGA-3’ |
|  | Reverse | 5’-CCCAGGAACTCGTGAAGGAC-3’ |
| *OPG* | Forward | 5’-CAAAGTAAACGCAGAGAGTGTAGA-3’ |
|  | Reverse | 5’-GAAGGTGAGGTTAGCATGTCC -3’ |
| *Actin* | Forward | 5’-CATCTCTTGCTCGAAGTCCA-3’ |
|  | Reverse | 5’-ATCATGTTTGAGACCTTCAACA-3’ |
